# Effect of Tasurgratinib as an Orally Available FGFR1–3 Inhibitor on Resistance to a CDK4/6 Inhibitor and Endocrine Therapy in ER^+^/HER2^−^ Breast Cancer Preclinical Models

**DOI:** 10.3390/cancers17071084

**Published:** 2025-03-24

**Authors:** Satoshi Kawano, Sayo Fukushima, Kyoko Nishibata, Ryu Gejima, Saori Watanabe Miyano

**Affiliations:** Eisai Co., Ltd., Tsukuba 300-2635, Japan; s-kawano@hhc.eisai.co.jp (S.K.); s3-fukushima@hhc.eisai.co.jp (S.F.); k-nishibata@hhc.eisai.co.jp (K.N.); r-gejima@hhc.eisai.co.jp (R.G.)

**Keywords:** tasurgratinib, FGFR, endocrine therapy, CDK4/6 inhibitor, breast cancer

## Abstract

Background: Fibroblast growth factor (FGF) signaling plays a crucial role in several cellular functions in cancer cells. Tasurgratinib, formerly known as E7090, is an orally available FGF receptor (FGFR)1–3 selective inhibitor. Here, we present the effects of tasurgratinib on the resistance to CDK4/6 inhibitors and endocrine therapy (ET) in a preclinical model. Methods: Estrogen receptor (ER)^+^ breast cancer (BC) patient-derived xenograft (PDX) models harboring *ESR1* wild-type or *ESR1* mutation were used as animal models. An in vitro cell proliferation assay of ER^+^ BC cell lines treated with fulvestrant or palbociclib + fulvestrant was conducted in the presence of FGF2 and FGF10, with or without tasurgratinib. Results: Among five ER^+^ BC PDX models, OD-BRE-0438 and OD-BRE-0704 showed higher sensitivities to tasurgratinib with prior palbociclib + fulvestrant than without it. In these models, palbociclib + fulvestrant treatment upregulated the expression of several *FGF* ligand mRNAs. In vitro, FGF2 and FGF10 decreased the sensitivity to both fulvestrant and palbociclib + fulvestrant, which was restored by co-treatment with tasurgratinib. Consistently, fulvestrant + tasurgratinib and elacestrant + tasurgratinib showed antitumor activity in ER^+^ BC PDX models harboring *ESR1* wild-type and *ESR1* mutation, respectively. In these models, fulvestrant or elacestrant upregulated the expression of several *FGF* ligand mRNAs. Conclusions: FGF signaling plays a role in resistance to CDK4/6 inhibitors and ET in ER^+^ BC. Tasurgratinib has the potential to exhibit significant antitumor activity in combination with ET against ER^+^ BC via FGF signaling inhibition. These findings indicate the therapeutic potential of tasurgratinib in treating ER^+^ BC.

## Simple Summary

Breast cancer is the most common cancer affecting women worldwide. Among breast cancer subtypes, estrogen receptor (ER)^+^/human epidermal growth factor receptor 2 (HER2)^−^ breast cancer is the most prevalent category, with a prevalence of 70%. For this type of tumor, a combination of endocrine therapy and a CDK4/6 inhibitor serves as the standard of care in advanced or metastatic cases. Here, we investigated the role of fibroblast growth factor (FGF) signaling in resistance to CDK4/6 inhibitors and endocrine therapy, as well as the effects of tasurgratinib, an FGFR1–3 selective inhibitor, on drug resistance in preclinical ER^+^/HER2^−^ breast cancer cell lines and patient-derived xenograft models. Our findings suggest that FGF signaling activation confers resistance to CDK4/6 inhibitors and endocrine therapy and that tasurgratinib shows antitumor activity in combination with endocrine therapy in endocrine therapy-resistant ER^+^ breast cancer.

## 1. Introduction

Breast cancer is the most common cancer affecting women worldwide, with incidence rates increasing each year. Within the spectrum of breast cancer subtypes, estrogen receptor (ER)^+^/human epidermal growth factor receptor 2 (HER2)^−^ breast cancer represents the most prevalent category, with a prevalence of 70% of all cases [1]. In this type of tumor, CDK4/6 inhibitors palbociclib, ribociclib, and abemaciclib have shown an improvement in progression-free survival (PFS) when combined with endocrine therapy (ET) [2,3,4]; therefore, a combination of ET and a CDK4/6 inhibitor serves as the standard of care in advanced or metastatic cases. Palbociclib, the first CDK4/6 inhibitor to receive FDA approval for patients with ER^+^/HER2^−^ breast cancer in combination with ET [5], has been the most widely used CDK4/6 inhibitor. Fulvestrant, a selective estrogen receptor degrader (SERD), is a new class of ET that degrades ER, reduces ER activity, and inhibits the growth of ER^+^ breast cancer [6]. However, real-world data indicate that second-line fulvestrant monotherapy after CDK4/6 inhibitor treatment provides suboptimal disease control [7,8], highlighting the need to develop improved treatment strategies to prolong the duration of effective ET-based treatments [9]. Elacestrant is also an SERD that degrades ERs and inhibits gene transcription, induction, and cell proliferation; however, unlike fulvestrant, it can be taken orally and has potent inhibitory activity against both wild-type ESR1 and mutant ESR1 [10]. Elacestrant received its first approval for use in patients with ER^+^/HER2^−^ breast cancer harboring mutant ESR1 [11].

Fibroblast growth factor (FGF) signaling plays a crucial role in the proliferation, survival, migration, and drug resistance of cancer cells as well as in tumor angiogenesis [12]. Moreover, gene alterations in *FGF/FGF receptor (FGFR)* have been reported after treatment with a CDK4/6 inhibitor and ET in patients with ER^+^ breast cancer, suggesting that FGF signaling mediates resistance to these therapies [13,14,15]. Tasurgratinib, formerly known as E7090, is an orally available FGFR1–3 selective inhibitor that belongs to a unique class of tyrosine kinase inhibitors, type V, with high affinity for target proteins and selectivity for a small number of receptor tyrosine kinases [16,17,18]. Tasurgratinib has been approved in Japan for the treatment of patients with unresectable biliary tract cancer with *FGFR2* fusion genes that progress after chemotherapy [19,20].

Here, we investigated the role of FGF signaling in resistance to CDK4/6 inhibitors or ET and the effects of tasurgratinib on drug resistance in preclinical ER^+^/HER2^−^ breast cancer cell lines and patient-derived xenograft (PDX) models. Our data suggest that the activation of FGF signaling confers resistance to CDK4/6 inhibitors and ET and that tasurgratinib shows antitumor activity in combination with ET in ET-resistant ER^+^ breast cancer. This study provides insights into the potential therapeutic impact of targeting FGF signaling in overcoming resistance to CDK4/6 inhibitors and ET in ER^+^ breast cancer, as further explored in the ongoing Phase 1 clinical trial of tasurgratinib in combination with endocrine therapies (NCT04572295) [21].

## 2. Materials and Methods

### 2.1. Test Agents

Tasurgratinib succinate (tasurgratinib) was synthesized by Eisai Co., Ltd. (Tsukuba, Japan) in accordance with patent publications WO 2014129477 (US 20140235614), WO 2016027781, US 20130338134, WO 2013108809, WO 2007071752, and WO 2007075869. Palbociclib HCl and palbociclib isethionate were purchased from Selleck Chemicals (Houston, TX, USA) for the in vivo studies. Palbociclib isethionate for in vitro studies was purchased from Changzhou Element Chemical Co., Ltd. (Changzhou, China). Fulvestrant for in vitro studies was purchased from MedChemExpress (Monmouth Junction, NJ, USA), and Faslodex^®^ for in vivo studies was purchased from AstraZeneca. Elacestrant dihydrochloride was purchased from MedChemExpress.

### 2.2. Animal Studies

Five established PDX models of ER^+^ breast cancer were supplied by Oncodesign Services (Dijon, France): OD-BRE-0438 (harboring D123Y in FGFR1; the effect on protein function is unknown), OD-BRE-0704 (harboring a synonymous variant of FGFR3 H290H [c.870C>T]), OD-BRE-0450, OD-BRE-0188, and IM-BRE-556. The additional characteristics of these five models are shown in File S1. Animal studies of these five models were conducted at Oncodesign Services using female athymic Swiss nude mice (Crl:NU(Ico)-*Foxn1*^nu^) from the Charles River Laboratories France (Saint-Germain Nuelles, France) or conducted at Eisai Co., Ltd. using female NOD SCID mice (NOD.CB17-*Prkdc*^scid^/J) from The Jackson Laboratory Japan, Inc. (Yokohama, Japan). Animal studies using two established PDX models of *ESR1* mutation-positive breast cancer, ST2056 (harboring Y537S in ESR1) and ST2535 (harboring D538G in ESR1), were conducted at XenoSTART (San Antonio, TX, USA). Animal studies on XenoSTART were conducted using female athymic nude mice (Crl:NU(NCr)-*Foxn1*^nu^) obtained from Charles River Laboratories (Wilmington, MA, USA). To ensure stable growth and engraftment, mice harboring OD-BRE-0438, OD-BRE-0704, OD-BRE-0450, OD-BRE-0188, and IM-BRE-556 received water continuously supplemented with 2.5 μg/mL estradiol. Regarding PDX models of *ESR1* mutation-positive breast cancer, the ST2056 model facilitates subcutaneous growth in mice without exogenous estradiol supplementation because of the ligand-independent activation of the mutated ERα [22]. However, the ST2535 model received water continuously supplemented with 6 μg/mL estradiol for passage, amplification, or experimentation according to the protocol in XenoSTART. The data of passage number of PDX in each experiment are shown in File S11.

Tasurgratinib was dissolved in distilled water or 10 mmol/L HCl and administered by oral gavage at 25 mg/kg. Palbociclib was dissolved with 0.9% NaCl or 50 mmol/L Na-lactate buffer (pH 4.0) and administered by oral gavage at 100 mg/kg. Fulvestrant (Faslodex^®^) was administered subcutaneously at 5 mg/mouse or 250 mg/kg. Elacestrant was dissolved with a 0.5% methylcellulose solution and administered by oral gavage at 30 mg/kg.

The tumor sizes and body weights were measured twice weekly. The tumor was measured in two dimensions, and the volume was calculated using the following formula: TV (mm^3^) = 1⁄2 length (mm) × [width (mm)]^2^.

The dT/C (% of control) for tumor growth and T/C (% of control) in each model were calculated using the following formula:

dT/C =
  $$\frac{\left(\mathrm{TV}\;\mathrm{on}\;\mathrm{Day}\;X\right)-\left(\mathrm{TV}\;\mathrm{on}\;\mathrm{Day}\;0\right)}{(\mathrm{mean}\;\mathrm{of}\;\mathrm{TV}\;\mathrm{in}\;\mathrm{control}\;\mathrm{group}\;\mathrm{on}\;\mathrm{Day}\;X)-(\mathrm{mean}\;\mathrm{of}\;\mathrm{TV}\;\mathrm{in}\;\mathrm{control}\;\mathrm{group}\;\mathrm{on}\;\mathrm{Day}\;0)}\times \;100$$
T/C =
  $$\frac{\left(\mathrm{TV}\;\mathrm{on}\;\mathrm{Day}\;X\right)}{(\mathrm{mean}\;\mathrm{of}\;\mathrm{TV}\;\mathrm{in}\;\mathrm{control}\;\mathrm{group}\;\mathrm{on}\;\mathrm{Day}\;X)}\times \;100$$
  where Day X means Day 14 for the OD-BRE-0438 model in the with-prior-treatment cohort; Day 21 for the OD-BRE-0438 model in the no-prior-treatment cohort, the OD-BRE-0704 model in the no-prior-treatment cohort, the OD-BRE-0450 model in both the no-prior-treatment cohort and with-prior-treatment cohort, and the OD-BRE-0188 model in the no-prior-treatment cohort; Day 23 for the OD-BRE-0188 model in the with-prior-treatment cohort; and Day 24 for the OD-BRE-0704 model in the with-prior-treatment cohort and the IM-BRE-556 model in both the no-prior-treatment cohort and with-prior-treatment cohort. The days were selected because of the first measurement after the end of the administration period or the final measurement in the control group. The values of T/C (% of control) were calculated for the OD-BRE-0188 and IM-BRE-556 models because the control groups for both models showed tumor regression after prior palbociclib + fulvestrant treatment, and the calculation of dT/C (% of control) for tumor growth was not applicable in such cases. The median PFS was assessed as the median time to a 1.5-fold increase in tumor volume from treatment initiation.

The effects of the indicated administration on tumor volume are shown as tumor volume relative to the initial tumor volume, and body weight is shown as body weight relative to the initial body weight. Data show the mean or the mean ± standard error of the mean (SEM). Differences in tumor volumes were analyzed using GraphPad Prism software version 10.4.1 (GraphPad Software, San Diego, CA, USA). Statistical significance was set at a *p*-value < 0.05 (two-sided). The data of tumor volume in each mouse before treatment period are shown in File S10.

### 2.3. Expression Analyses

Tumor samples were collected on the day after the two-week administration of palbociclib (100 mg/kg, once daily [Q1D] × 14) + fulvestrant (5 mg/mouse, once a week [Q7D] × 2), fulvestrant (250 mg/kg, Q7D × 2), or elacestrant (30 mg/kg, Q1D × 15). Tumor tissues were excised, and RNA was extracted and purified using the Maxwell RSC SimplyRNA Tissue Kit (Promega, Madison, WI, USA). Purified total RNA was reverse transcribed using a High Capacity cDNA Reverse Transcription Kit (Thermo Fisher Scientific, Waltham, MA, USA). TaqMan Fast Advanced Master Mix (Thermo Fisher Scientific) and TaqMan Gene Expression Assays (Hs99999909_m1 for *HPRT1*, Hs01092738_m1 for *FGF1*, Hs00940253_m1 for *FGF7*, Hs00171832_m1 for *FGF8*, Hs00610298_m1 for *FGF10*, Hs00182599_m1 for *FGF17*, Hs00241111_m1 for *FGFR1*, Hs01552926_m1 for *FGFR2*, and Hs00179829_m1 for *FGFR3*; Thermo Fisher Scientific) were used for quantitative RT-PCR (q-PCR), which was conducted on a QuantStudio™ 7 Flex Real-Time PCR System (Thermo Fisher Scientific). The threshold cycle (Ct) values for each sample were measured in duplicates, and the mean Ct values were used for analysis. Changes in gene expression were assessed by comparing the delta Ct values for each sample, with *HPRT1* as a housekeeping gene. Data show the geometric mean (n < 3) or the geometric mean ± geometric standard deviation (SD; n ≥ 3). Differences in gene expression levels were analyzed using the GraphPad Prism software (GraphPad Software). Statistical significance was set at a *p*-value < 0.05 (two-sided).

### 2.4. In Vitro Antiproliferation Assay

Cell lines were obtained from ECACC (MCF7 and ZR-75-1) and ATCC (HCC1428). MCF7 cells were maintained in Eagle’s minimum essential medium (FUJIFILM Wako, Osaka, Japan) containing 10% (*v*/*v*) heat-inactivated fetal bovine serum (FBS) (Sigma-Aldrich, St. Louis, MO, USA). ZR-75-1 and HCC1428 cells were maintained in RPMI1640 (FUJIFILM Wako) containing 10% (*v*/*v*) heat-inactivated FBS. Cell line identities were verified by STR fingerprinting, and mycoplasma contamination was ruled out using the Venor^®^GeM Advance (Minerva Biolabs, Skillman, NJ, USA) or MycoAlert kit (Lonza, Basel, Switzerland). These cancer cell lines were seeded in 96-well plates at a density of 1.0 × 10^3^ cells/well for MCF7 (Figure 3a), 1.5 × 10^3^ cells/well for MCF7 (Figure 3b), or 2 × 10^3^ cells/well for others in the medium as described above (Day 1). The next day, FGF stimulation using 25 ng/mL FGF2 (Thermo Fisher Scientific) and 100 ng/mL FGF10 (R&D Systems, Minneapolis, MN, USA) to induce FGF signaling activation, treatment with palbociclib/fulvestrant, and co-treatment with 100 nmol/L tasurgratinib to inhibit the FGF signaling pathway were initiated simultaneously (Day 2). Medium with or without cytokines/drugs was refreshed every 3–4 d. The viable cell number was determined using CellTiter-Glo 2.0 (Promega) when the control cells reached approximately 90% confluence (Figure 3a: Day 9 for MCF7, Day 14 for ZR-75-1, and Day 16 for HCC1428; Figure 3b: Day 7 for MCF7, Day 10 for ZR-75-1, and Day 15 for HCC1428). The measurement was performed in triplicates, and data show the ratio to control (without fulvestrant or without palbociclib + fulvestrant in each culture condition; mean ± SD) calculated using Spotfire^®^ version 7.11 (TIBCO Software, Santa Clara, CA, USA).

### 2.5. Statistical Analyses

**Figure 1:**
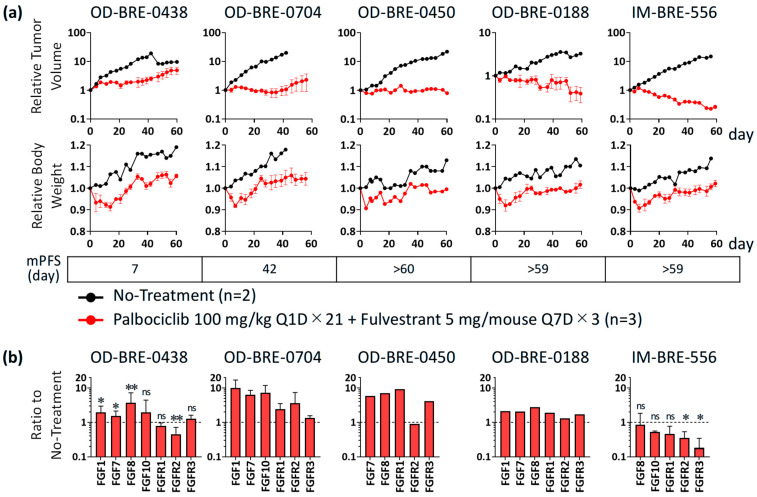
Antitumor activity and gene expression alterations of FGF/FGFR with palbociclib + fulvestrant in five ER^+^ breast cancer PDX models (OD-BRE-0438, OD-BRE-0704, OD-BRE-0450, OD-BRE-0188, IM-BRE-556). (**a**) Relative tumor volume (upper) and relative body weight (lower) after treatment with palbociclib (100 mg/kg, once daily [Q1D] × 21) in combination with fulvestrant (5 mg/mouse, once a week [Q7D] × 3). Data show the mean (no treatment groups: n = 2 per group) or the mean ± SEM (palbociclib + fulvestrant treatment groups: n = 3 per group). (**b**) Alterations of human mRNA expression in tumors collected on the day following the treatment with palbociclib (100 mg/kg, Q1D × 14) in combination with fulvestrant (250 mg/kg, Q7D × 2). Data show the geometric mean (OD-BRE-0450 and OD-BRE-0188: n = 2 per group) or the geometric mean ± geometric SD (OD-BRE-0438: n = 6 per group, OD-BRE-0704 and IM-BRE-556: n = 3 per group). The expressions of FGF1 in OD-BRE-0450 and IM-BRE-556, FGF7 in IM-BRE-556, FGF8 in OD-BRE-0704, and FGF10 in OD-BRE-0450 and OD-BRE-0188 were not detected in both baseline and post-treatment. * *p* < 0.05, ** *p* < 0.01 versus the no treatment group (unpaired *t*-test) in the OD-BRE-0438 and IM-BRE-556 models in which mouse numbers were more than two in both no treatment groups and treated groups. Values of Ct and delta Ct, *p*-values in unpaired *t*-test, and ratio to no treatment are shown in File S2. mPFS: median progression-free survival; ns: not significant.

**Figure 2:**
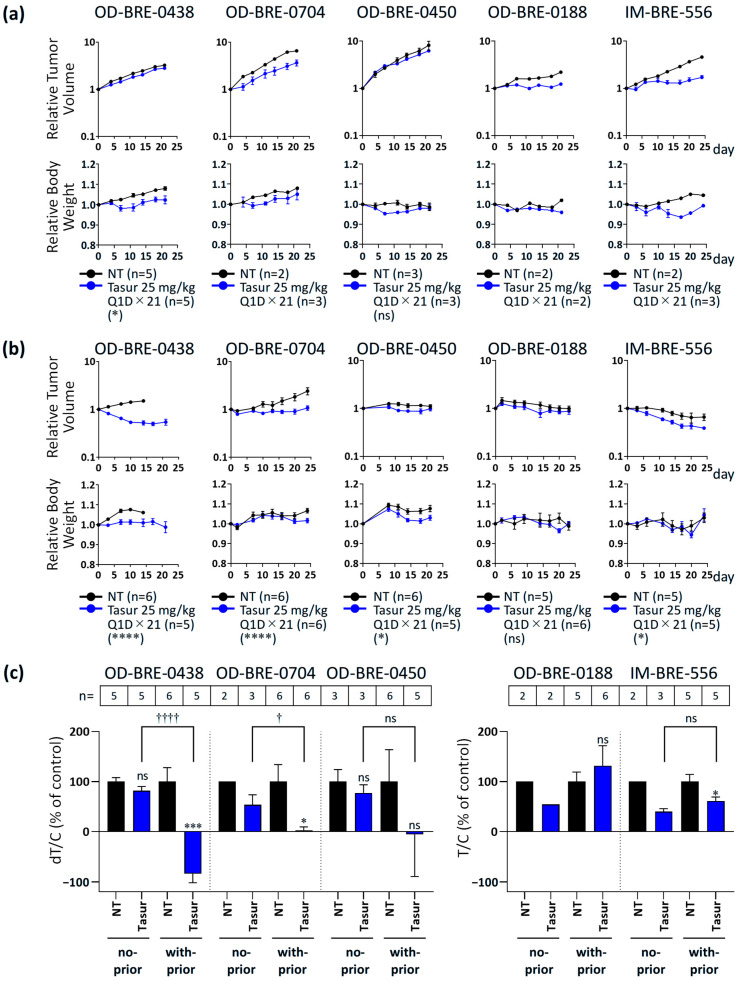
Antitumor activity of tasurgratinib with or without prior palbociclib + fulvestrant treatment in five ER^+^ breast cancer PDX models (OD-BRE-0438, OD-BRE-0704, OD-BRE-0450, OD-BRE-0188, and IM-BRE-556). Relative tumor volume (upper) and relative body weight (lower) after the treatment with tasurgratinib (25 mg/kg, once daily [Q1D] × 14 or Q1D × 21) with no prior treatment (**a**) or with prior treatment with palbociclib (100 mg/kg, Q1D × 14) in combination with fulvestrant (250 mg/kg, once a week × 2) (**b**). * *p* < 0.05, **** *p* < 0.0001 versus the no treatment group (repeated measures analysis of variance). (**c**) dT/C (% of control) for tumor growth in models of OD-BRE-0438, OD-BRE-0704, and OD-BRE-0450, and T/C (% of control) in the OD-BRE-0188 and IM-BRE-556 models. * *p* < 0.05, *** *p* < 0.001 versus the no treatment group (unpaired *t* test), † *p* < 0.05, †††† *p* < 0.0001 the no-prior-treatment cohort versus with-prior-treatment cohort (unpaired t test). Values of dT/C (% of control) for tumor growth in models of OD-BRE-0438, OD-BRE-0704, and OD-BRE-0450, and T/C (% of control) values in the OD-BRE-0188 and IM-BRE-556 models are shown in File S3. The numbers of mice used in each group were indicated within the figure. Data show the mean (n < 3 per group) or the mean ± SEM (n ≥ 3 per group). ns: not significant, NT: no treatment, Tasur: tasurgratinib.

**Figure 3:**
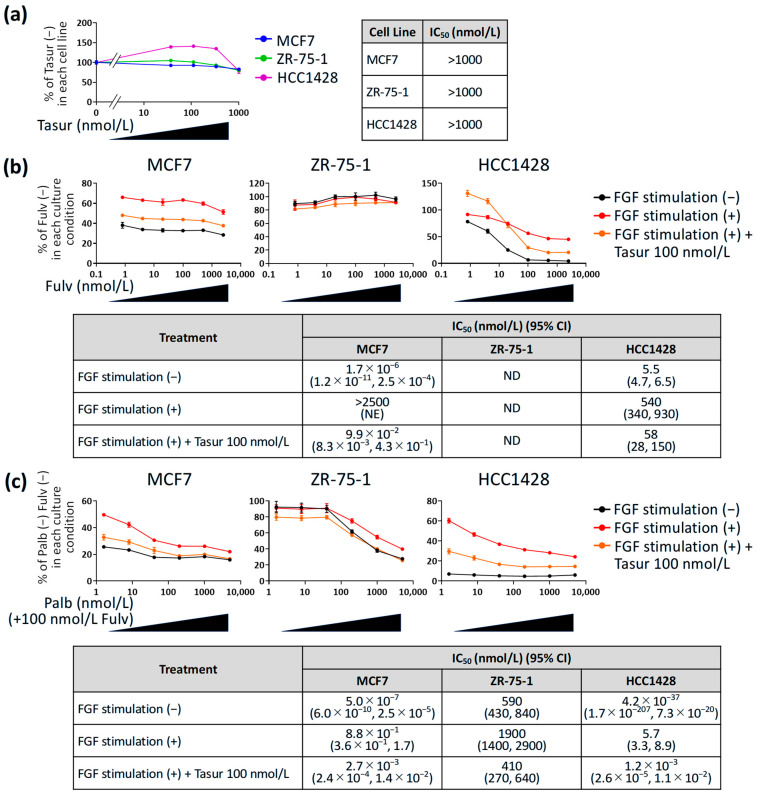
Anti-proliferative activity of tasurgratinib, fulvestrant, or palbociclib + fulvestrant against ER^+^ breast cancer cell lines with FGF stimulation in vitro. (**a**) In vitro study to analyze the effect of tasurgratinib on cell growth in the presence of 25 ng/mL FGF2 and 100 ng/mL FGF10 in ER^+^ breast cancer cell lines, MCF7, ZR-75-1, and HCC1428. (**b**,**c**) In vitro study to analyze the effect of FGF stimulation using 25 ng/mL FGF2 and 100 ng/mL FGF10 with or without tasurgratinib on cell growth inhibitory activities of fulvestrant (**b**) or palbociclib + fulvestrant (100 nmol/L) combination (**c**) in ER^+^ breast cancer cell lines, MCF7, ZR-75-1, and HCC1428. Data show the ratio to control (without tasurgratinib [a], without fulvestrant [b], or without palbociclib + fulvestrant [c] in each culture condition; mean ± SD; n = 3 per group). Values of ratio to control are shown in File S4. CI: confidence interval, Fulv: fulvestrant, IC_50_: 50% inhibitory concentration, ND: not determined, NE: not estimable, Palb: palbociclib, Tasur: tasurgratinib.

**Figure 4:**
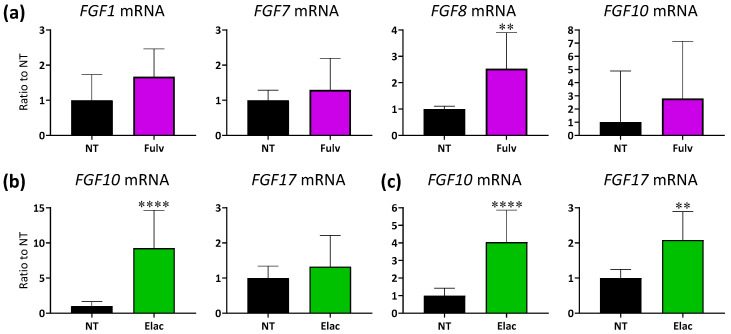
Human mRNA expression in tumors collected the day after the two-week treatment. (**a**) Treatment with fulvestrant (250 mg/kg, once a week × 2) in the OD-BRE-0438 model. (**b**) Treatment with elacestrant (30 mg/kg, once daily [Q1D] × 15) in the ST2056 model. (**c**) Treatment with elacestrant (30 mg/kg, Q1D × 15) in the ST2535 model. Data show the geometric mean ± geometric SD (n = 5 per group for OD-BRE-0438, n = 6 per group for ST2056 and ST2535). ** *p* < 0.01, **** *p* < 0.0001 versus the no treatment group (unpaired *t*-test). Values of Ct and delta Ct, *p*-values in unpaired *t*-test, and ratio to no treatment are shown in File S5. Elac: elacestrant, Fulv: fulvestrant, NT: no treatment.

**Figure 5:**
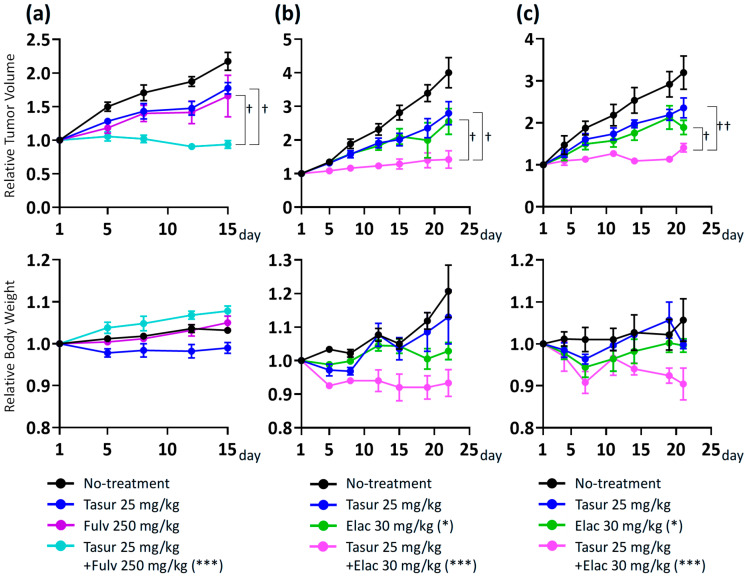
Combination activity of tasurgratinib on fulvestrant or elacestrant in ER^+^ breast cancer PDX models. Relative tumor volume (upper) and relative body weight (lower) are shown. (**a**) Treatment with tasurgratinib (25 mg/kg, once daily [Q1D] × 14) or fulvestrant (250 mg/kg, once a week [Q7D] × 2) or both in the OD-BRE-0438 model. (**b**) Treatment with tasurgratinib (25 mg/kg, Q1D × 21) or elacestrant (30 mg/kg, Q1D × 21) or both in the ST2056 model. (**c**) Treatment with tasurgratinib (25 mg/kg, Q1D × 21) or elacestrant (30 mg/kg, Q1D × 21) or both in the ST2535 model. Data show the mean ± SEM (n = 5 per group for the OD-BRE-0438 model, n = 6 per group for the ST2056 model, and n = 6 per group [no treatment group and tasurgratinib group] or n = 5 per group [elacestrant group and combination group] for the ST2535 models). * *p* < 0.05, *** *p* < 0.001 versus the no treatment group, † *p* < 0.05, †† *p* < 0.01 versus the combination group (one-way analysis of variance [ANOVA] followed by Dunnett’s multiple comparison test by using relative tumor volumes on Day 15 in the OD-BRE-0438 model. Repeated measures ANOVA followed by Dunnett’s multiple comparison test in the ST2056 and ST2535 models). Elac: elacestrant, Fulv: fulvestrant, Tasur: tasurgratinib.

All animal studies were performed in biological duplicates or more, as shown in each figure legend. The in vitro study in Figure 3 was performed in biological triplicates. Statistical analyses of the treated group versus the no treatment group in Figure 1b were performed using the unpaired *t*-test. Statistical analyses of the treated group versus the no treatment group (Figure 2a,b) were performed using repeated measures analysis of variance (ANOVA). Statistical analyses of the treated group versus the no treatment group or the no-prior-treatment cohort versus the with-prior-treatment cohort (Figure 2c) were performed using an unpaired *t*-test. The 50% inhibitory concentration (IC_50_) values and 95% confidence intervals (CI) were generated from sigmoidal curves representing the degree of growth inhibition versus compound concentrations of experiments performed in biological triplicates. Statistical analyses of the treated group versus the no treatment group (Figure 4) were performed using an unpaired *t*-test. Statistical analyses of the treated group versus the no treatment group and the mono-treated group versus the combination group (Figure 5) were performed using one-way ANOVA followed by Dunnett’s multiple comparison test by using relative tumor volumes on Day 15 in the OD-BRE-0438 model and repeated measures ANOVA followed by Dunnett’s multiple comparison test in the ST2056 and ST2535 models. *p*-values were calculated using the GraphPad Prism software (GraphPad Software). Statistical significance was set at a *p*-value < 0.05 (two-sided).

## 3. Results

### 3.1. Antitumor Activity and Alterations of FGF/FGFR Expression with Palbociclib + Fulvestrant Treatments in ER^+^ Breast Cancer PDX Models

ER^+^ breast cancer patients with high FGFR1 mRNA expression at baseline show resistance to a CDK4/6 inhibitor + letrozole and had a poor prognosis in the MONALEESA-2 study [14]. Furthermore, FGF/FGFR gene alterations were observed in 24 of 60 patients (40%) following CDK4/6 inhibitor and ET treatment [13]. These data suggest that FGF signaling mediates resistance to these therapies. Initially, the antitumor activities of palbociclib, a CDK4/6 inhibitor, in combination with fulvestrant, an ET, were analyzed in five established PDX models of ER^+^ breast cancer. Among these, OD-BRE-0438, OD-BRE-0704, and OD-BRE-0450 showed relatively low sensitivity (less tumor shrinkage) to palbociclib + fulvestrant (Figure 1a). To provide molecular mechanistic insights into the differences in sensitivity, gene expression alterations were analyzed using tumor samples collected on the day following the two-week administration period of palbociclib (100 mg/kg, Q1D × 14) + fulvestrant (5 mg/mouse, Q7D × 2). The OD-BRE-0438 model with the most resistant phenotype, as evidenced by the shortest median PFS, showed statistically significant upregulated gene expression of FGF1, FGF7, and FGF8 ligands in cancer cells, while FGFR1, FGFR2, or FGFR3 were not upregulated (Figure 1b). The other PDX models with relatively low sensitivity (OD-BRE-0704 and OD-BRE-0450) also tended to show upregulated gene expression of several FGF ligands in cancer cells (Figure 1b). In contrast, the OD-BRE-0188 and IM-BRE-556 models showed relatively high sensitivity with tumor shrinkage to palbociclib + fulvestrant, in which notable upregulation of FGF ligand gene expression was not observed. These results suggest that FGF signaling plays an important role in resistance to CDK4/6 inhibitors and ET treatments, as previously reported in clinical data [13,14]. Our copy number (CN) variation analysis indicated that OD-BRE-0704 and IM-BRE-0556 exhibit FGFR1 gene amplification with positivity criteria of CN ≥ 6 [23], and gene amplifications of FGFR2 and FGFR3 were not detected in all models with positivity criteria of CN > 5 for FGFR2 [24] and CN > 7 for FGFR3 [25]. This analysis also indicated FGF3/4/19 amplification in the OD-BRE-0450 and OD-BRE-0188 models. Notably, the status of CN variations in each model was not altered after the two-week administration of palbociclib + fulvestrant (Supplementary Table S1; Supplementary Materials and Methods are shown in File S1). These data suggest that the important role of FGF signaling in resistance to CDK4/6 inhibitors and ET treatments is independent of the amplification status of each gene. Of particular importance in understanding the FGF signaling-related resistance mechanism is the finding that upregulating the expression levels of FGF ligand mRNA, rather than FGFR mRNA or gene alterations, is critical.

### 3.2. Antitumor Activity of Tasurgratinib with or Without Prior Palbociclib + Fulvestrant Treatment in ER^+^ Breast Cancer PDX Models

The upregulated expression of several FGF ligand genes after palbociclib + fulvestrant treatment prompted us to analyze the changes in the dependency of cancer cells on FGF signaling, with or without prior palbociclib + fulvestrant treatment, in the five established PDX models of ER^+^ breast cancer. Tasurgratinib treatment delayed tumor growth in all ER^+^ breast cancer PDX models tested (Figure 2a). However, OD-BRE-0438 and OD-BRE-0704 showed higher sensitivity to tasurgratinib with prior palbociclib + fulvestrant treatment than without it (Figure 2b,c). These results suggest that the inhibition of CDK4/6 in combination with ET pushes breast cancer cells toward an alternative FGF signaling dependence to sustain cellular proliferation via a CDK4/6- and endocrine-independent mechanism.

### 3.3. Effect of Tasurgratinib on Anti-Proliferative Activity of Fulvestrant or Palbociclib + Fulvestrant Against ER^+^ Breast Cancer Cell Lines with FGF Stimulation In Vitro

Subsequently, the effect of FGF stimulation on the anti-proliferative activity of fulvestrant and palbociclib + fulvestrant was evaluated in vitro using three ER^+^ breast cancer cell lines: MCF7, ZR-75-1, and HCC1428. In these cell lines, Ct values below 35 in q-PCR suggested detectable expressions of FGFR1–3 (Supplementary Table S2; Individual Ct values are shown in File S9). Furthermore, western blotting confirmed phosphorylation of FGFR (Tyr653/654), FGFR substrate 2 (FRS2)-α (Tyr436), an adapter protein identified as a major downstream mediator of FGF signaling, and ERK1/2 (Thr202/Tyr204) in these cell lines in the presence of 25 ng/mL FGF2 and 100 ng/mL FGF10 (Supplementary Figure S1; Supplementary Materials and Methods are shown in File S1, uncropped blots are shown in File S6, and values of band intensities are shown in File S7). Hence, we chose 25 ng/mL FGF2 and 100 ng/mL FGF10 as a method to stimulate FGF signaling in subsequent experiments. First, we confirmed that tasurgratinib alone showed no effect on the growth of these cell lines, even in the presence of FGF ligands (Figure 3a), although we proved that tasurgratinib at 10 nmol/L or more inhibits FGF signaling in multiple cell lines in vitro [17,18]. Based on our findings, we selected 100 nmol/L as a concentration of tasurgratinib to inhibit FGF signaling without cytotoxic effects on ER^+^ breast cancer cell lines. In MCF7 and HCC1428, the presence of FGF ligands decreased the anti-proliferative activity of fulvestrant (Figure 3b). Furthermore, stimulation decreased the anti-proliferative activity of the palbociclib + fulvestrant combination in all three cell lines (Figure 3c). In contrast, co-treatment with tasurgratinib tended to restore the anti-proliferative activity of fulvestrant in MCF7 and HCC1428 cells, as well as that of palbociclib + fulvestrant in all three cell lines (Figure 3b,c). These results suggest that FGF signaling developed resistances of cultured ER^+^ breast cancer cells to fulvestrant and the CDK4/6 inhibitor palbociclib.

### 3.4. Expression Levels of FGF Ligand mRNA in ER^+^ Breast Cancer PDX Tumors with ET Treatment

Further experiments were conducted using fulvestrant monotreatment in an ER^+^ breast cancer PDX model to allow for more clinically relevant studies on second-line fulvestrant monotherapy. This is because fulvestrant monotherapy provides suboptimal disease control in patients with breast cancer [7,8], and we confirmed that FGF stimulation confers resistance to the palbociclib + fulvestrant combination and fulvestrant monotherapy (Figure 3). We selected the OD-BRE-0438 model for further study because it exhibited the most robust change in sensitivity to tasurgratinib with and without prior palbociclib + fulvestrant treatments (Figure 2). This suggests that among the five ER^+^ breast cancer PDX models, FGF signaling plays the strongest role as a resistance factor in this model. The expression levels of FGF ligand mRNA in tumors tended to be upregulated after treatment with fulvestrant for two weeks in this model (Figure 4a). Elacestrant is also an SERD that degrades ERs and exhibits potent inhibitory activity against both wild-type ESR1 and mutant ESR1 [10]. Hence, changes in expression were analyzed after treatment with elacestrant for two weeks in ER^+^ breast cancer PDX models of ST2056 harboring Y537S in ESR1 and ST2535 harboring D538G in ESR1. Treatment with elacestrant tended to upregulate the mRNA expression levels of FGF ligands in both models (Figure 4b,c). Furthermore, alterations in FGFR1–3 were also analyzed in these models. Treatment with fulvestrant did not alter FGFR1–3 expression in the OD-BRE-0438 model. Elacestrant treatment upregulated and downregulated FGFR3 expression in the ST2056 and ST2535 models, respectively. In contrast, FGFR1 upregulation was observed in the ST2535 model after treatment with elacestrant, and the treatment downregulated FGFR2 expression in the ST2056 model (Supplementary Figure S2).

### 3.5. Combination Activity of Tasurgratinib on Fulvestrant or Elacestrant in ER^+^ Breast Cancer PDX Models

Finally, to investigate the possibility of combining the antitumor activity of the FGFR inhibitor with ET, further in vivo experiments were conducted using PDX models. Fulvestrant in combination with tasurgratinib showed antitumor activity superior to that of each monotherapy in the OD-BRE-0438 model (Figure 5a). Furthermore, elacestrant in combination with tasurgratinib showed superior antitumor activity compared to that of each monotherapy in both the ST2056 and ST2535 models (Figure 5b,c).

## 4. Discussion

FGF7/FGFR2 signaling induces resistance to tamoxifen via the PI3K/AKT pathway, with Ser167 phosphorylation in the ER [26]. FGF signaling has also been reported to push cells toward alternative CDK4/6- and endocrine-independent mechanisms [27,28]. Moreover, FGFR2 overactivation reportedly results in cross-resistance between SERD and CDK inhibitors [13,14]. Based on the data, targeting FGF signaling was discussed as a novel therapeutic strategy to overcome resistance during ER^+^/HER2^−^ breast cancer treatment [29].

Here, we confirmed that FGF signaling activation confers resistance to a CDK4/6 inhibitor and ET in ER^+^/HER2^−^ breast cancer preclinical models, as previously reported. Furthermore, tasurgratinib, an FGFR1–3 selective inhibitor, and fulvestrant, an ET, showed antitumor activity in preclinical models of ET-resistant ER^+^ breast cancer. In addition, tasurgratinib showed antitumor activity in combination with elacestrant, a next-generation oral SERD showing significant PFS benefit in patients with *ESR1*-mutated breast cancer [30] in two ER^+^ breast cancer PDX models harboring *ESR1* mutations. These models exhibited a tendency of upregulation of *FGF* ligand mRNA expression in tumors, suggesting that the activation of FGF signaling confers resistance to ET in ER*^+^*/HER2^−^ breast cancer. These data suggest that tasurgratinib has the potential to re-sensitize ET-resistant ER^+^ breast cancer cells to ET. The gene amplification in *FGFR1* has been shown to confer resistance to CDK4/6 inhibitor + letrozole and leads to a poor prognosis in patients with ER^+^ breast cancer [14]. To date, several selective FGFR inhibitors have been tested in clinical trials in patients with ER^+^ breast cancer harboring *FGFR1* amplification, but sensitivity to FGFR inhibitors has not been determined by *FGFR1* amplification [31,32]. These results suggest that gene amplifications in *FGFR* may be insufficient to change the dependency of ER^+^ breast cancer cells on FGF signaling. In fact, our data indicated that FGF signaling activation, leading to resistance to CDK4/6 inhibitors and ET, occurs by upregulating the expression levels of *FGF* ligand mRNA rather than gene alterations. In addition, alterations in *FGFR* mRNA expression after treatment with a CDK4/6 inhibitor or ET were not consistent in our study (Supplementary Figure S2; Values of Ct and delta Ct, *p*-values in unpaired *t*-test, and ratio to no treatment are shown in File S8), underscoring the importance of the expression levels of *FGF* ligand mRNA in resistance to these treatments. Our study did not analyze FGF signaling status in such situations; therefore, developing a method to detect FGF signaling activation, such as a gene signature score, is warranted for clinical application.

Our in vitro cell proliferation assay showed that MCF7 and HCC1428 exhibited a change in sensitivity to fulvestrant in the presence of FGF ligands, whereas ZR-75-1 did not. Compared with MCF7 or HCC1428, ZR-75-1 had less sensitivity to fulvestrant even in the absence of FGF ligands. The data were consistent with those of a previous report [33], suggesting that ZR-75-1 may have a resistance mechanism to fulvestrant that is independent of FGF signaling. Therefore, stimulation with FGF ligand had no effect. Further analysis of the characteristics of the tested cell lines, such as multi-omics analysis, may provide new insights to determine the biomarker to select suitable patients for FGFR inhibitor and ET combination.

Of particular importance is the combined antitumor activity of tasurgratinib with not only fulvestrant, an earlier generation SERD, but also a next-generation oral SERD. Several new SERDs are currently under development to overcome the limitations of the existing SERD, including its poor activity against *ESR1* mutation-positive breast cancer and the necessity of intramuscular injection because of its poor bioavailability [10]. In January 2023, elacestrant was the first oral SERD to receive FDA approval for the treatment of patients with *ESR1*-mutated ER^+^/HER2^−^ metastatic breast cancer [34]. Furthermore, imlunestrant, another next-generation oral SERD, showed prolonged PFS compared to standard therapy among patients with *ESR1*-mutated ER*^+^*/HER2^−^ metastatic breast cancer [35]. Considering these findings and our preclinical evidence, tasurgratinib is a promising treatment option as a combination therapy for ET.

This study had several limitations. First, we used a small number of PDX and cell lines as preclinical models. This approach allowed us to determine the combined antitumor activity of tasurgratinib with ET in three PDX models, OD-BRE-0438, ST2056, and ST2535; however, it remains unclear whether these findings can be generalized to a broader population. In addition, potential combined antitumor activity was not analyzed in PDX models where ET did not induce upregulation of several *FGF* ligand genes. Second, alterations in signaling pathways other than FGF signaling after treatment with a CDK4/6 inhibitor or ET were not explored. Several resistance mechanisms have been reported for CDK4/6 inhibitors and ET, such as the Cyclin E/CDK2 axis, mitogen-activated protein kinase (MAPK) pathway alterations, and tumor microenvironment alterations [36,37]; therefore, a precise understanding of the other resistance mechanisms would help predict preclinical models or populations to show the antitumor activity of tasurgratinib in combination with ET with high precision.

A Phase 1 study of tasurgratinib as monotherapy or in combination with fulvestrant or exemestane for patients with ER^+^/HER2^−^ breast cancer is ongoing (NCT04572295) [21]. Our findings provide preclinical evidence supporting the use of tasurgratinib in combination with SERD in patients with ER^+^/HER2^−^ breast cancer. Additionally, promising preliminary antitumor activity of tasurgratinib in combination with fulvestrant has been observed in patients with ER*^+^*/HER2^−^ breast cancer [21,38]. Further studies are warranted to address the medical need for improved treatment strategies to extend the duration of effective ET-based treatment [9] because second-line fulvestrant monotherapy after CDK4/6 inhibitor treatment provides suboptimal disease control [7,8].

## 5. Conclusions

In this study, we investigated the effects of tasurgratinib, an FGFR1–3 selective inhibitor, on drug resistance to a CDK4/6 inhibitor and ET in preclinical models. These data suggest that activation of FGF signaling plays a role in resistance to a CDK4/6 inhibitor and ET against ER^+^ breast cancer and that tasurgratinib exhibits significant antitumor activity in combination with ET against ER^+^ breast cancer based on inhibition of the FGF signaling pathway.

## Data Availability

The data presented in this study are available in this article and the associated Supplementary Materials accessible online. Further inquiries can be directed to the authors.

## Supplementary Materials

The following supporting information can be downloaded at: https://www.mdpi.com/article/10.3390/cancers17071084/s1. Figure S1: The effect of FGF stimulation in three ER^+^ breast cancer cell lines, MCF7, ZR-75-1, and HCC1428; Figure S2: Human mRNA expression in tumors collected the day after the two-week treatment; Table S1: Predicted copy numbers of each gene in five ER^+^ breast cancer PDX models (OD-BRE-0438, OD-BRE-0704, OD-BRE-0450, OD-BRE-0188, and IM-BRE-556); Table S2: The Ct values of *FGFR1*, *FGFR2*, *FGFR3*, and *HPRT1* in three ER^+^ breast cancer cell lines, MCF7, ZR-75-1, and HCC1428; File S1: Supplementary Materials and Methods; File S2: Values of Ct and delta Ct, *p*-values in unpaired *t*-test, and ratio to no treatment in Figure 1b; File S3: Values of dT/C (% of control) for tumor growth in models of OD-BRE-0438, OD-BRE-0704, and OD-BRE-0450, and T/C (% of control) values in the OD-BRE-0188 and IM-BRE-556 models in Figure 2c; File S4: Values of ratio to control in Figure 3; File S5: Values of Ct and delta Ct, *p*-values in unpaired *t*-test, and ratio to no treatment in Figure 4; File S6: The original images of uncropped Western blot of Figure S1a; File S7: Values of band intensities in Figure S1b; File S8: Values of Ct and delta Ct, *p*-values in unpaired *t*-test, and ratio to no treatment in Figure S2; File S9: Individual Ct values in Table S2, File S10: Tumor volume in each mouse before treatment period; File S11: Passage number of PDX in each experiment.
