# Supplementary material for: Effect of Tasurgratinib as an Orally Available FGFR1–3 Inhibitor on Resistance to a CDK4/6 Inhibitor and Endocrine Therapy in ER+/HER2− Breast Cancer Preclinical Models
Source: Cancers (Basel). 2025 Mar 24;17(7):1084. doi: 10.3390/cancers17071084 (PMC11988047; doi:10.3390/cancers17071084)
Supplement: Supplementary file 1 [file cancers-17-01084-s001.zip › cancers-3491685-supplementary.pdf]

cancers

MDPI

Supplementary Materials

Effect of Tasurgratinib as an Orally Available FGFR1-3 Inhibitor on Resistance to a CDK4/6 Inhibitor and Endocrine Therapy in ER+/HER2− Breast Cancer Preclinical Models

Satoshi Kawano [1], Sayo Fukushima [1], Kyoko Nishibata [1], Ryu Gejima [1], Saori Watanabe Miyano [1,*]

[1] Eisai Co., Ltd., Tsukuba, Japan
* Correspondence: s5-watanabe@hhc.eisai.co.jp; Tel.: +81 298475900

Figure S1: The effect of FGF stimulation in three ER+ breast cancer cell lines, MCF7, ZR-75-1, and HCC1428.

Figure S2: Human mRNA expression in tumors collected the day after the two-week treatment.

Table S1: Predicted copy numbers of each gene in five ER+ breast cancer PDX models (OD-BRE-0438, OD-BRE-0704, OD-BRE-0450, OD-BRE-0188, and IM-BRE-556).

Table S2: The Ct values of *FGFR1*, *FGFR2*, *FGFR3*, and *HPRT1* in three ER+ breast cancer cell lines, MCF7, ZR-75-1, and HCC1428.

File S1: Supplementary Materials and Methods.

File S2: Values of Ct and delta Ct, *p*-values in unpaired t test, and ratio to no-treatment in Figure 1b.

File S3: Values of dT/C (% of control) for tumor growth in models of OD-BRE-0438, OD-BRE-0704, and OD-BRE-0450, and T/C (% of control) values in the OD-BRE-0188 and IM-BRE-556 models in Figure 2c.

File S4: Values of ratio to control in Figure 3.

File S5: Values of Ct and delta Ct, *p*-values in unpaired t test, and ratio to no-treatment in Figure 4.

File S6: The original images of uncropped Western blot of Figure S1a.

File S7: Values of band intensities in Figure S1b.

File S8: Values of Ct and delta Ct, *p*-values in unpaired t test, and ratio to no-treatment in Figure S2.

File S9: Individual Ct values in Table S2.

File S10: Tumor volume in each mouse before treatment period.

File S11: Passage number of PDX in each experiment.

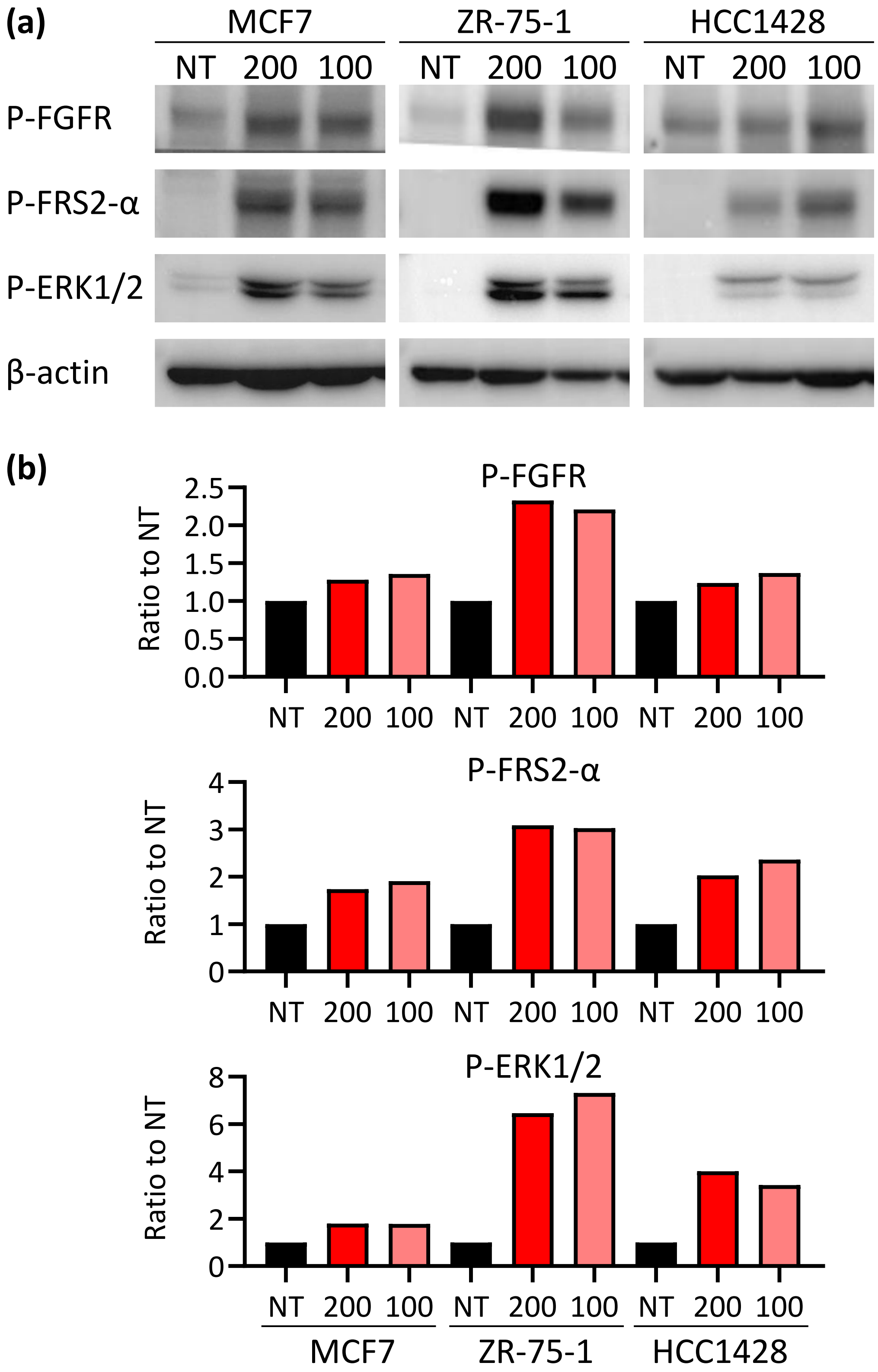

Figure S1. The effect of FGF stimulation in three ER^+ breast cancer cell lines, MCF7, ZR-75-1, and HCC1428. (**a**) Western blot images of phosphorylation of FGFR (Tyr653/654) and its downstream molecules, phosphorylation of FGFR substrate 2 (FRS2)-α (Tyr436), and phosphorylation of ERK1/2 (Thr202/Tyr204). β-Actin served as loading control. The original images of uncropped Western blot and molecular weight markers are shown in the Supplementary File S1. (**b**) The bands were quantified and normalized to β-actin and presented as ratio to no-treatment. NT: no-treatment, 200: treatment with 25 ng/mL bFGF + 200 ng/mL FGF10 for 30 min, 100: treatment with 25 ng/mL bFGF + 100 ng/mL FGF10 for 30 min.

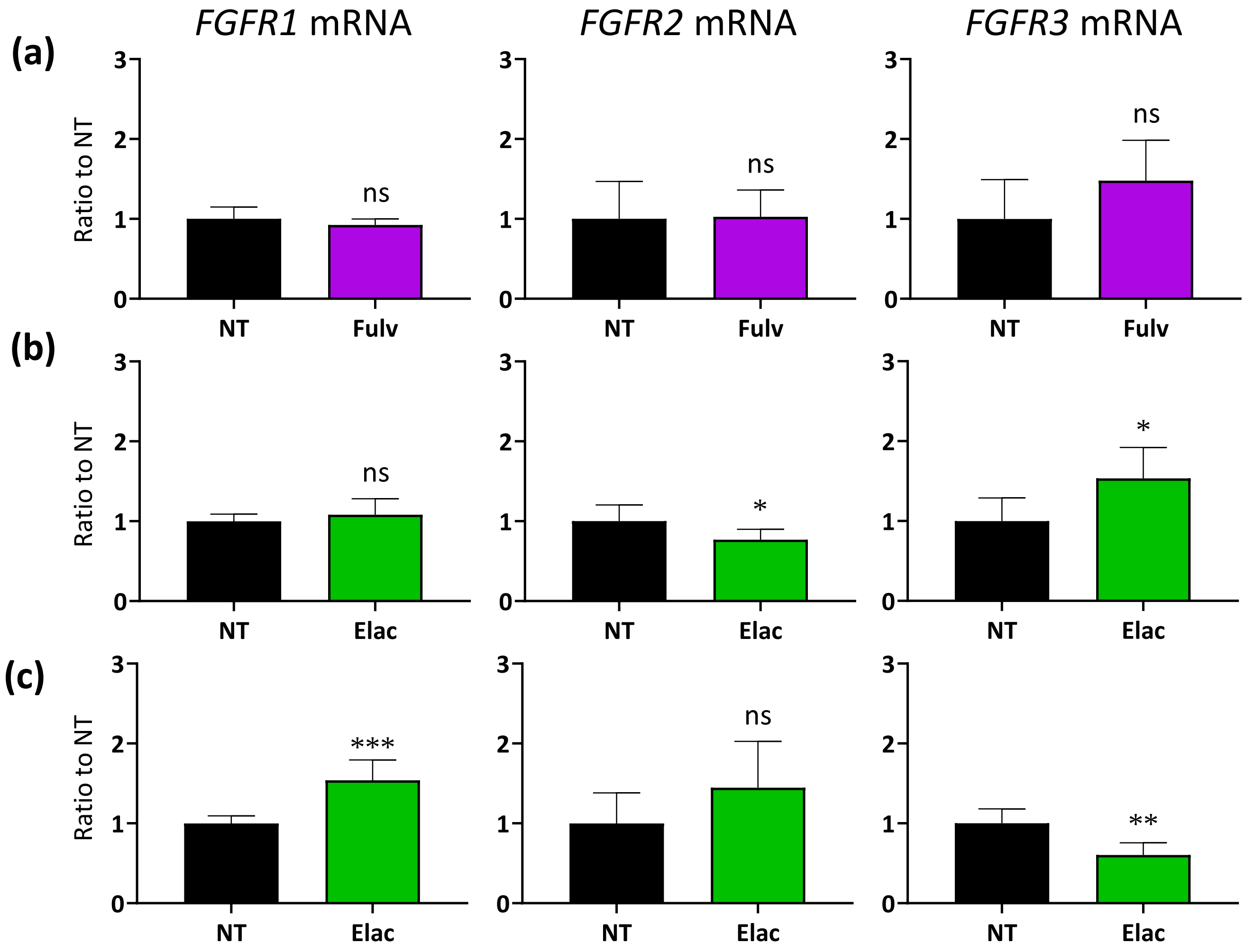

Figure S2. Human mRNA expression in tumors collected the day after the two-week treatment. (**a**) Treatment with fulvestrant (250 mg/kg, once a week × 2) in the OD-BRE-0438 model. (**b**) Treatment with elacestrant (30 mg/kg, once daily [Q1D] × 15) in the ST2056 model. (**c**) Treatment with elacestrant (30 mg/kg, Q1D × 15) in the ST2535 model. Data show the geometric mean ± geometric SD (n=5 per group for OD-BRE-0438, n=6 per group for ST2056 and ST2535). *p<0.05, **p<0.01, and ***p<0.001 versus the no-treatment group (unpaired t test). Elac: elacestrant, Fulv: fulvestrant, NT: no-treatment.

Table S1. Predicted copy numbers of each gene in five ER^+ breast cancer PDX models (OD-BRE-0438, OD-BRE-0704, OD-BRE-0450, OD-BRE-0188, and IM-BRE-556).

Model	Sample	*FGF3*	*FGF4*	*FGF19*	*FGFR1*	*FGFR2*	*FGFR3*
OD-BRE-0438	No-treatment	2	1	1	2	2	2
	Palb+Fulv for 2 weeks	2	2	2	2	2	2
OD-BRE-0704	No-treatment	3	2	2	29	3	2
	Palb+Fulv for 2 weeks	4	3	2	31	3	2
OD-BRE-0450	No-treatment	9	6	5	2	2	1
	Palb+Fulv for 2 weeks	10	6	6	2	2	1
OD-BRE-0188	No-treatment	4	3	3	4	2	2
	Palb+Fulv for 2 weeks	6	4	3	4	2	2
IM-BRE-556	No-treatment	2	2	2	12	3	1
	Palb+Fulv for 2 weeks	3	2	2	15	3	2

In groups of Palb+Fulv for 2 weeks, tumor samples were collected on the next day after the treatment with palbociclib (100 mg/kg, once daily × 14) in combination with fulvestrant (5 mg/mouse for OD-BRE-0438, OD-BRE-0704, and OD-BRE-0450, or 250 mg/kg for OD-BRE-0188 and IM-BRE-556, once a week × 2).

Table S2. The Ct values of *FGFR1*, *FGFR2*, *FGFR3*, and *HPRT1* in three ER^+ breast cancer cell lines, MCF7, ZR-75-1, and HCC1428.

Cell Line	***FGFR1***		***FGFR2***		***FGFR3***		***HPRT1***	
	**Mean**	**SD**	**Mean**	**SD**	**Mean**	**SD**	**Mean**	**SD**
MCF7	29.3	0.4	31.9	0.3	29.1	0.2	28.5	0.5
ZR-75-1	26.4	0.5	30.1	0.3	30.1	0.2	30.3	0.3
HCC1428	29.2	0.3	28.6	0.2	32.4	0.3	29.6	0.3

HPRT1 was used as a housekeeping gene. The values were determined by q-PCR and data show the mean and SD (n=3 per group).

File S1. Supplementary Materials and Methods.

Copy number variation (CNV) analysis

The presence of CNV was analyzed in genomic DNA from each patient-derived xenograft collected before or after the two-week administration of palbociclib (100 mg/kg, once daily×14) + fulvestrant (5 mg/mouse for OD-BRE-0438, OD-BRE-0704, and OD-BRE-0450, or 250 mg/kg for OD-BRE-0188 and IM-BRE-556, once a week×2) using an Applied Biosystems TaqMan Copy Number Assay (Thermo Fisher Scientific). The CNV assay (Hs02862256_cn for *FGFR1*, Hs05182482_cn for *FGFR2*, Hs03518314_cn for *FGFR3*, Hs06336027_cn for *FGF3*, Hs01235235_cn for *FGF4*, and Hs00147838_cn for *FGF19*) were used for quantitative RT-PCR, which was conducted on a QuantStudio™ 7 Flex Real-Time PCR Systems (Thermo Fisher Scientific). Data was analyzed using CopyCaller™ Software (v2.1, Thermo Fisher Scientific).

Western blotting

MCF7 cells were seeded in 6-well plates in Eagle's minimum essential medium (FUJIFILM Wako) containing 10% (v/v) heat-inactivated fetal bovine serum (FBS; Sigma-Aldrich). ZR-75-1 and HCC1428 cells were seeded in 6-well plates with RPMI1640 (FUJIFILM Wako) containing 10% (v/v) heat-inactivated FBS. The next day, FGF stimulation using 25 ng/mL FGF2 (Thermo Fisher Scientific) and 100 ng/mL or 200 ng/mL FGF10 (R&D Systems) was performed for 30 min to activate FGF signaling. Collected cell lysates were subjected to western blot analysis. The blots were developed using the Immobilon Western Chemiluminescent HRP Substrate (Millipore, Burlington, MA, USA), and chemiluminescence was detected using an image analyzer FUSION-FX7-826 (Vilber, Collégien, France). Antibodies against phospho-FGFR (Tyr653/654), phospho-FRS2-α (Tyr436), and phospho-ERK1/2 (Thr202/Tyr204) were obtained from Cell Signaling Technology (Danvers, MA, USA). Antibody against β-actin was obtained from Sigma-Aldrich. The band intensities were quantified using Fusion Capt Advance FX7 software (version 17.04a; Vilber).

The characteristics of the OD-BRE-0438 model

Model Category: **Luminal B breast cancer model**

Patient clinical annotations:

Sex: F

Age: 51

Tumor sample origin: Breast

Primary/Metastasis/Recurrence: Primary

Histology:

Invasive ductal carcinoma

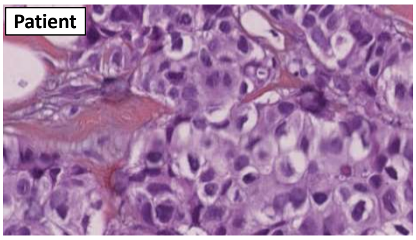

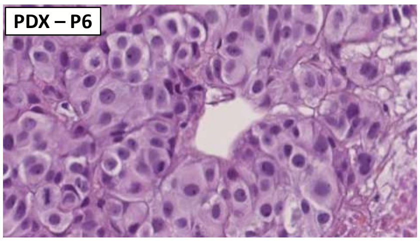

PDX markers:

ER +++, PR ++, HER2 -, AR ++

In vivo tumor growth for master passage: P6

Host strain: SCID

Estrogen support: Yes

Graft site: SC

Time to reach 100 mm³: 38 days

Time to reach 1000 mm³: 56 days

In vivo tumor growth for model thawing:

Recommended host strain for model thawing: *NOG*

Tumor take rate (%): 100

Time to reach 200 mm³: 50 days

In vivo tumor growth curves:

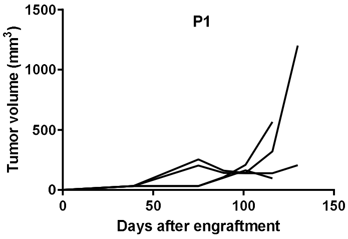

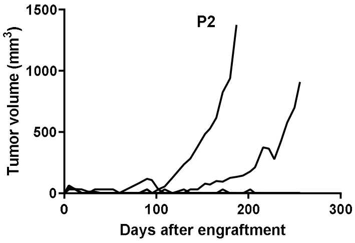

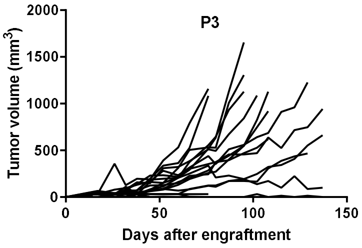

The characteristics of the OD-BRE-0704 model

Model Category: **Luminal B breast cancer model**

Patient clinical annotations:

Sex: F

Age: 73

Tumor sample origin: Breast

Primary/Metastasis/Recurrence: Primary

Histology:

Invasive ductal carcinoma

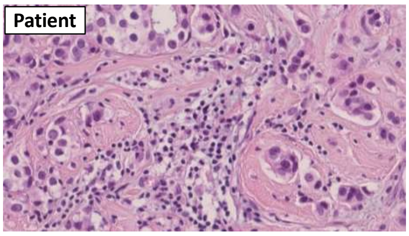

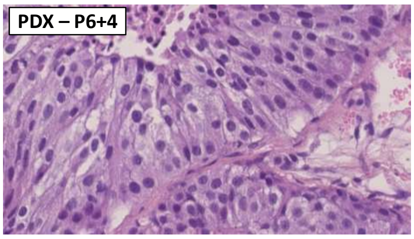

PDX markers:

ER +++, PR +++, HER2 -, AR -

In vivo tumor growth for master passage: P10 (6+4)

Host strain: BALB/c Nude

Estrogen support: Yes

Graft site: SC

Time to reach 100 mm^3: 71 days

Time to reach 1000 mm^3: 91 days

In vivo tumor growth for model thawing:

Recommended host strain for model thawing: *NOG*

Tumor take rate (%): *25*

Time to reach 200 mm^3: 107 days

In vivo tumor growth curves:

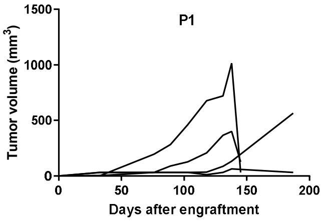

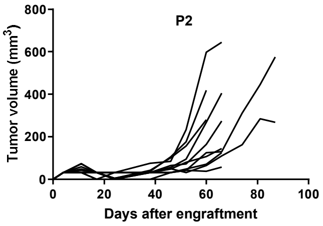

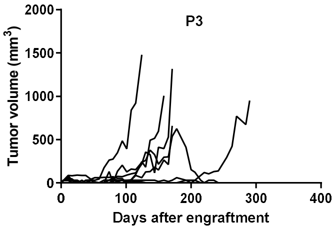

The characteristics of the OD-BRE-0450 model

Model Category: **Luminal B breast cancer model**

Patient clinical annotations:

Sex: F

Age: 38

Tumor sample origin: Breast

Primary/Metastasis/Recurrence: Primary

Histology:

Invasive ductal lobular carcinoma

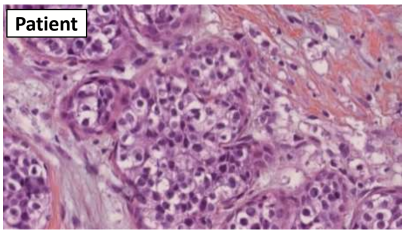

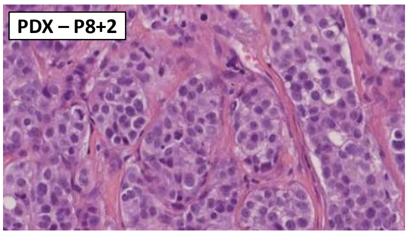

PDX markers:

ER ++, PR +, HER2 -, AR -

In vivo tumor growth for master passage: P8

Host strain: BALB/c Nude

Estrogen support: Yes

Graft site: SC

Tumor take rate (%):

Time to reach 100 mm^3: 44 days

Time to reach 1000 mm^3: 61 days

In vivo tumor growth for model thawing:

Recommended host strain for model thawing: NOG

Tumor take rate (%): 86

Time to reach 200 mm^3: 83 days

In vivo tumor growth curves:

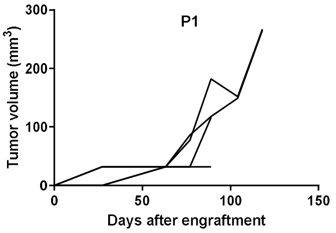

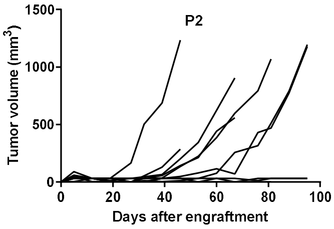

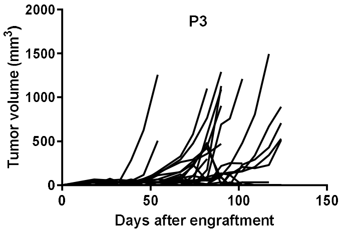

The characteristics of the OD-BRE-0188 model

Model Category: **Luminal A breast cancer model**

Patient clinical annotations:

Sex: F

Age: 65

Tumor sample origin: Breast

Primary/Metastasis/Recurrence: Primary

Histology:

Poorly differentiated invasive ductal carcinoma with lymph node metastases

Patient

Not available

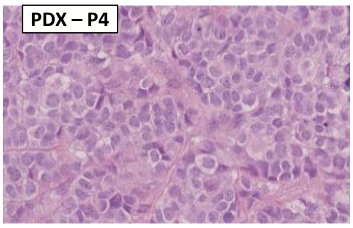

PDX markers:

ER ++, PR +++, HER2 -, AR +

In vivo tumor growth for master passage: P4

Host strain: Swiss Nude

Estrogen support: Yes

Graft site: SC

Time to reach 100 mm³: 25 days

Time to reach 1000 mm³: 115 days

In vivo tumor growth for model thawing:

Recommended host strain for model thawing: *NOG*

Tumor take rate (%): 90

Time to reach 200 mm³: 75 days

In vivo tumor growth curves:

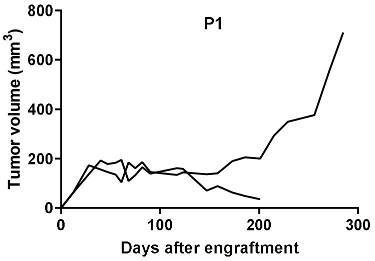

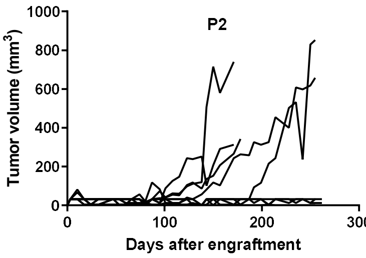

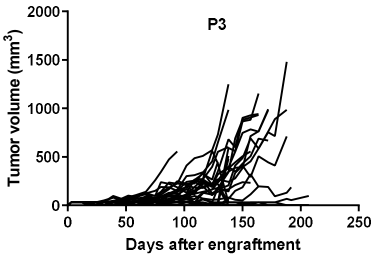

The characteristics of the IM-BRE-556 model

Model Category: **Luminal breast cancer model**

Patient clinical annotations:

Sex: F

Age: 76

Tumor sample origin: Breast

Primary/Metastasis/Recurrence: Primary

Histology:

Ductal breast carcinoma poorly differentiated

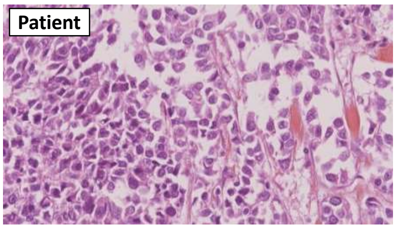

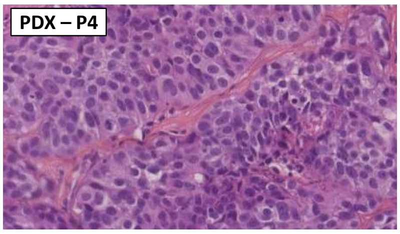

PDX markers:

ER +++; PR ++; HER2 -; AR -

In vivo tumor growth for master passage: P4

Host strain: BALB/c Nude

Estrogen support: Yes

Graft site: SC

Time to reach 100 mm^3: 49 days

Time to reach 1000 mm^3: 98 days

In vivo tumor growth for model thawing:

Recommended host strain for model thawing: NSG

Tumor take rate (%): *83*

Time to reach 200 mm^3: 95 days

In vivo tumor growth curves:

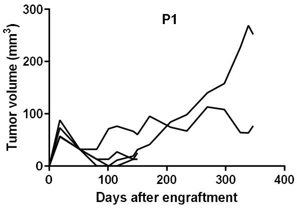

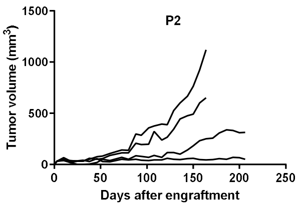

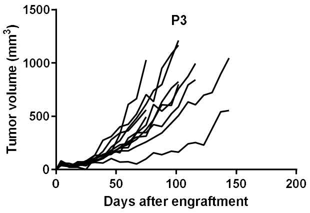

File S2. Values of Ct and delta Ct, *p* -values in unpaired t test, and ratio to no-treatment in Figure 1b.

Model	Gene	Treatment	Ct Value						Delta Ct Value							Ratio to No-Treatment					
															Unpaired t test						
OD-BRE-0438	*HPRT1*	NT	25.133	25.002	24.893	25.067	25.136	24.562													
		Palb+Fulv	24.931	24.962	24.437	24.607	25.391	25.069													
	FGF1	NT	35.637	35.855	36.749	35.725	37.038	35.760	10.505	10.853	11.856	10.657	11.901	11.198	0.0190						
		Palb+Fulv	34.532	35.464	34.989	34.455	34.985	36.125	9.601	10.502	10.553	9.848	9.594	11.056		2.95	1.58	1.53	2.49	2.96	1.08
	FGF7	NT	33.540	32.625	33.900	33.193	33.698	32.988	8.407	7.623	9.007	8.125	8.561	8.427	0.0489						
		Palb+Fulv	32.088	33.080	32.749	32.178	32.649	33.128	7.156	8.118	8.313	7.571	7.258	8.060		2.30	1.18	1.03	1.73	2.14	1.23
	FGF8	NT	34.143	33.488	34.433	34.284	33.859	33.613	9.011	8.486	9.540	9.217	8.722	9.052	0.0013						
		Palb+Fulv	31.818	32.487	33.026	31.599	30.965	32.211	6.887	7.525	8.590	6.992	5.574	7.142		4.34	2.79	1.33	4.04	10.79	3.64
	FGF10	NT	24.193	25.015	25.035	24.590	24.556	26.306	-0.940	0.014	0.142	-0.478	-0.580	1.744	0.1500						
		Palb+Fulv	24.094	23.847	25.481	23.919	23.082	23.075	-0.837	-1.115	1.045	-0.688	-2.309	-1.994		1.77	2.14	0.48	1.59	4.90	3.94
	FGFR1	NT	27.750	27.181	27.705	27.632	27.488	26.188	2.618	2.180	2.812	2.565	2.352	1.626	0.1288						
		Palb+Fulv	27.748	27.228	27.435	27.599	27.917	27.688	2.817	2.266	2.999	2.992	2.526	2.619		0.73	1.07	0.64	0.64	0.89	0.83
	FGFR2	NT	25.671	25.241	25.820	25.498	25.713	25.134	0.539	0.239	0.927	0.431	0.577	0.572	0.0023						
		Palb+Fulv	26.784	27.032	26.911	26.491	26.012	26.344	1.853	2.071	2.474	1.884	0.621	1.275		0.40	0.35	0.26	0.40	0.95	0.60
	FGFR3	NT	28.157	27.445	27.367	27.933	28.187	27.211	3.025	2.443	2.474	2.866	3.051	2.650	0.0650						
		Palb+Fulv	26.869	27.291	27.214	27.220	27.487	27.671	1.938	2.329	2.777	2.613	2.096	2.603		1.76	1.34	0.98	1.10	1.58	1.11
OD-BRE-0704	*HPRT1*	NT	24.402	24.536	-	-	-	-													
		Palb+Fulv	26.287	25.963	25.401	-	-	-													
	FGF1	NT	32.616	32.646	-	-	-	-	8.214	8.110	-	-	-	-							
		Palb+Fulv	30.525	30.534	31.134	-	-	-	4.239	4.571	5.733	-	-	-		15.17	12.05	5.39	-	-	-
	FGF7	NT	32.729	32.671	-	-	-	-	8.327	8.135	-	-	-	-							
		Palb+Fulv	31.661	31.978	30.652	-	-	-	5.375	6.015	5.250	-	-	-		7.24	4.65	7.89	-	-	-
	FGF10	NT	29.280	27.206	-	-	-	-	4.878	2.671	-	-	-	-							
		Palb+Fulv	27.166	27.604	25.618	-	-	-	0.879	1.641	0.217	-	-	-		7.44	4.39	11.77	-	-	-
	FGFR1	NT	24.279	24.139	-	-	-	-	-0.123	-0.397	-	-	-	-							
		Palb+Fulv	24.494	24.064	24.458	-	-	-	-1.792	-1.899	-0.944	-	-	-		2.89	3.11	1.61	-	-	-
	FGFR2	NT	31.789	31.713	-	-	-	-	7.387	7.177	-	-	-	-							
		Palb+Fulv	31.060	30.857	32.034	-	-	-	4.773	4.894	6.632	-	-	-		5.69	5.23	1.57	-	-	-
	FGFR3	NT	28.451	28.687	-	-	-	-	4.049	4.151	-	-	-	-							
		Palb+Fulv	29.729	29.689	29.275	-	-	-	3.442	3.726	3.874	-	-	-		1.58	1.30	1.17	-	-	-
OD-BRE-0450	*HPRT1*	NT	30.152	29.820	-	-	-	-													
		Palb+Fulv	32.276	32.758	-	-	-	-													
	FGF7	NT	35.044	34.592	-	-	-	-	4.893	4.772	-	-	-	-							
		Palb+Fulv	34.724	34.822	-	-	-	-	2.448	2.064	-	-	-	-		5.22	6.81	-	-	-	-
	FGF8	NT	36.378	36.660	-	-	-	-	6.226	6.841	-	-	-	-							
		Palb+Fulv	36.564	35.826	-	-	-	-	4.288	3.068	-	-	-	-		4.74	11.05	-	-	-	-
	FGFR1	NT	27.762	27.888	-	-	-	-	-2.390	-1.931	-	-	-	-							
		Palb+Fulv	27.366	26.933	-	-	-	-	-4.911	-5.825	-	-	-	-		6.73	12.68	-	-	-	-
	FGFR2	NT	29.443	29.353	-	-	-	-	-0.708	-0.467	-	-	-	-							
		Palb+Fulv	32.255	31.938	-	-	-	-	-0.021	-0.820	-	-	-	-		0.68	1.18	-	-	-	-
	FGFR3	NT	30.492	30.515	-	-	-	-	0.340	0.695	-	-	-	-							
		Palb+Fulv	30.991	30.940	-	-	-	-	-1.285	-1.818	-	-	-	-		3.49	5.05	-	-	-	-
OD-BRE-0188	*HPRT1*	NT	30.055	28.741	31.828	-	-	-													
		Palb+Fulv	30.348	31.143	-	-	-	-													
	FGF1	NT	33.558	34.241	35.845	-	-	-	3.502	5.500	4.017	-	-	-							
		Palb+Fulv	34.472	33.502	-	-	-	-	4.124	2.359	-	-	-	-		1.16	3.95	-	-	-	-
	FGF7	NT	34.273	32.824	36.265	-	-	-	4.218	4.083	4.437	-	-	-							
		Palb+Fulv	33.993	33.904	-	-	-	-	3.645	2.761	-	-	-	-		1.52	2.80	-	-	-	-
	FGF8	NT	34.847	34.888	36.645	-	-	-	4.792	6.147	4.817	-	-	-							
		Palb+Fulv	34.794	34.302	-	-	-	-	4.446	3.159	-	-	-	-		1.75	4.26	-	-	-	-
	FGFR1	NT	25.979	27.125	29.441	-	-	-	-4.076	-1.616	-2.387	-	-	-							
		Palb+Fulv	26.663	27.569	-	-	-	-	-3.685	-3.573	-	-	-	-		1.99	1.84	-	-	-	-
	FGFR2	NT	29.196	29.388	31.140	-	-	-	-0.859	0.647	-0.688	-	-	-							
		Palb+Fulv	29.953	30.166	-	-	-	-	-0.395	-0.977	-	-	-	-		1.07	1.60	-	-	-	-
	FGFR3	NT	29.415	30.482	32.902	-	-	-	-0.640	1.741	1.074	-	-	-							
		Palb+Fulv	30.724	30.701	-	-	-	-	0.376	-0.441	-	-	-	-		1.27	2.24	-	-	-	-
IM-BRE-556	*HPRT1*	NT	28.996	32.686	35.084	30.386	-	-													
		Palb+Fulv	28.006	27.906	30.097		-	-													
	FGF8	NT	34.301	36.864	37.168	35.072	-	-	5.304	4.178	2.084	4.686	-	-	0.815						
		Palb+Fulv	33.042	32.761	33.126	-	-	-	5.036	4.854	3.029	-	-	-		0.51	0.58	2.05	-	-	-
	FGF10	NT	26.869	29.336	30.959	28.094	-	-	-2.127	-3.349	-4.125	-2.292	-	-	0.155						
		Palb+Fulv	25.916	25.974	28.000	-	-	-	-2.091	-1.932	-2.097	-	-	-		0.54	0.49	0.55	-	-	-
	FGFR1	NT	26.949	29.847	30.331	27.518	-	-	-2.047	-2.839	-4.753	-2.868	-	-	0.206						
		Palb+Fulv	26.211	26.505	27.262	-	-	-	-1.796	-1.402	-2.836	-	-	-		0.40	0.30	0.82	-	-	-
	FGFR2	NT	27.035	30.133	31.976	28.764	-	-	-1.962	-2.553	-3.108	-1.622	-	-	0.026						
		Palb+Fulv	27.711	27.244	28.639	-	-	-	-0.296	-0.662	-1.459	-	-	-		0.25	0.32	0.55	-	-	-
	FGFR3	NT	31.263	33.075	34.703	32.465	-	-	2.267	0.390	-0.382	2.079	-	-	0.038						
		Palb+Fulv	32.606	30.984	33.074	-	-	-	4.599	3.078	2.976	-	-	-		0.09	0.25	0.27	-	-	-

File S3. Values of dT/C (% of control) for tumor growth in models of OD-BRE-0438, OD-BRE-0704, and OD-BRE-0450, and T/C (% of control) values in the OD-BRE-0188 and IM-BRE-556 models in Figure 2c.

Model	Treatment		Tumor Volume		dT/C
			Day 0	Day 21	
OD-BRE-0438	no-prior	No-treatment	128	443	93
			134	407	81
			146	511	108
			159	473	93
			190	619	126
		Tasurgratinib	133	397	78
			137	309	51
			152	502	103
			153	449	87
			190	487	88
	Treatment		Day 0	Day 14	dT/C
	with-prior	No-treatment	148	203	37
			208	260	35
			231	362	86
			280	437	103
			291	469	118
			511	846	221
		Tasurgratinib	179	73	-70
			223	115	-71
			242	162	-53
			273	163	-73
			390	158	-153

Model	Treat		Tumor Volume		dT/C
			Day 0	Day 21	
OD-BRE-0704	no-prior	No-treatment	218	1448	136
			106	686	64
		Tasurgratinib	226	1057	92
			121	351	25
			168	567	44
	Treatment		Day 0	Day 24	dT/C
	with-prior	No-treatment	135	135	0
			298	799	247
			73	286	105
			98	270	85
			147	237	44
			157	397	118
		Tasurgratinib	105	94	-5
			201	231	15
			93	148	27
			214	175	-19
			123	121	-1
			193	195	1

Model	Treat		Tumor Volume		dT/C
			Day 0	Day 21	
OD-BRE-0450	no-prior	No-treatment	171	1102	61
			204	2396	143
			276	1761	97
		Tasurgratinib	166	914	49
			210	1402	78
			294	1909	105
	Treatment		Day 0	Day 21	dT/C
	with-prior	No-treatment	104	144	261
			117	125	52
			108	115	46
			55	42	-85
			151	200	320
			148	149	7
		Tasurgratinib	196	165	-202
			193	230	241
			88	110	143
			125	110	-98
			71	54	-111

Model	Treatment		Tumor Volume	T/C
			Day 21	
OD-BRE-0188	no-prior	No-treatment	230	61
			521	139
		Tasurgratinib	135	36
			275	73
	Treatment		Day 23	T/C
	with-prior	No-treatment	133	160
			47	57
			77	93
			55	66
			103	124
		Tasurgratinib	122	147
			68	82
			57	69
			267	322
			50	60
			92	111

Model	Treatment		Tumor Volume	T/C
			Day 24	
IM-BRE-556	no-prior	No-treatment	586	97
			620	103
		Tasurgratinib	272	45
			167	28
			285	47
	Treatment		Day 24	T/C
	with-prior	No-treatment	87	66
			146	110
			120	91
			110	83
			198	150
		Tasurgratinib	56	42
			101	76
			77	58
			109	82
			63	48

File S4. Values of ratio to control in Figure 3.

Ratio to control without tasurgratinib

Cell Line	Concentration of Tasurgratinib (nmol/L)	FGF stimulation (+)		
MCF7	0	95.23	101.84	102.93
	37	91.60	91.68	94.31
	111	90.75	93.72	93.21
	333	89.25	89.06	90.32
	1000	82.97	82.93	82.40
ZR-75-1	0	98.91	101.92	99.17
	37	104.39	105.35	104.57
	111	99.38	104.44	99.03
	333	93.24	92.04	93.34
	1000	79.46	82.31	77.70
HCC1428	0	103.50	100.57	95.93
	37	142.37	140.03	135.66
	111	142.26	144.29	136.91
	333	136.25	136.01	132.38
	1000	73.02	77.32	85.69

Ratio to control without fulvestrant in each culture condition

Cell Line	Concentration of Fulvestrant (nmol/L)	FGF stimulation (-)			FGF stimulation (+)			FGF stimulation (+) + Tasurgratinib 100 nmol/L		
MCF7	0.8	35.34	41.22	37.84	66.24	66.48	65.35	46.59	49.61	48.04
	4	32.28	33.87	35.41	63.41	61.85	64.14	43.82	45.63	44.99
	20	31.12	33.98	33.88	60.17	64.68	58.73	44.71	44.30	43.65
	100	31.44	32.50	34.02	63.68	63.54	62.99	43.86	44.02	43.52
	500	31.99	33.72	33.62	59.05	58.51	61.73	40.98	43.28	43.71
	2500	27.96	29.71	27.57	49.13	51.16	53.75	37.98	38.67	36.22
ZR-75-1	0.8	85.55	90.94	92.10	81.71	96.31	84.40	80.56	80.39	83.32
	4	88.21	92.86	92.41	88.50	89.17	87.49	82.53	83.48	85.21
	20	96.91	100.78	102.13	94.96	99.44	95.88	87.36	86.07	92.77
	100	94.06	103.56	103.23	100.40	100.02	97.17	86.76	89.37	93.96
	500	97.25	103.84	105.70	94.35	99.40	95.80	88.64	90.83	93.14
	2500	96.57	99.71	93.27	89.79	93.87	90.72	88.61	92.26	92.12
HCC1428	0.8	78.34	80.47	76.19	90.29	94.04	90.44	128.43	127.27	137.74
	4	63.44	56.14	61.19	86.89	83.29	89.53	112.32	122.28	115.29
	20	24.31	25.22	25.65	73.17	73.80	76.44	67.02	71.76	77.62
	100	6.95	6.19	6.38	56.47	56.06	56.47	27.48	30.85	29.79
	500	5.82	5.75	4.93	46.42	45.53	47.25	20.77	21.38	19.16
	2500	4.39	4.05	4.61	43.25	46.35	45.49	19.91	20.76	21.09

Ratio to control without palbociclib + fulvestrant in each culture condition

Cell Line	Concentration of Palbociclib (nmol/L)	FGF stimulation (-)			FGF stimulation (+)			FGF stimulation (+) + Tasurgratinib 100 nmol/L		
MCF7	1.6	25.56	26.60	24.67	50.13	48.61	50.19	31.67	31.48	35.36
	8	23.35	22.49	24.07	41.33	41.01	44.41	27.48	29.45	30.89
	40	17.24	18.07	18.05	30.82	29.33	31.68	20.64	22.79	25.41
	200	17.15	17.26	17.42	26.70	25.06	26.78	19.09	17.75	19.76
	1000	17.94	19.15	17.83	26.94	26.12	25.28	19.89	20.33	19.69
	5000	16.08	16.24	15.50	21.86	22.25	21.96	15.55	17.18	17.37
ZR-75-1	1.6	87.19	89.36	100.64	86.25	91.96	95.03	77.58	77.62	84.25
	8	87.59	89.43	98.14	83.49	93.61	92.26	75.65	78.22	82.00
	40	85.31	93.59	91.69	84.72	95.73	92.68	78.74	78.12	82.37
	200	64.13	61.63	59.86	73.03	78.27	74.24	55.92	56.72	59.21
	1000	36.10	40.20	37.57	52.14	56.07	56.41	40.04	40.71	39.87
	5000	27.78	27.83	27.76	38.24	40.90	40.64	24.21	26.40	26.49
HCC1428	1.6	7.44	6.86	6.97	61.12	62.24	57.66	30.84	26.68	31.67
	8	6.57	5.99	5.84	48.51	45.51	45.50	21.32	22.40	25.60
	40	5.74	4.84	5.15	36.77	36.93	36.74	17.06	17.17	16.16
	200	4.78	4.90	4.82	31.53	31.45	30.92	13.52	14.08	15.07
	1000	4.95	4.93	5.20	28.11	28.71	28.05	14.50	13.76	15.21
	5000	6.39	6.24	5.59	25.05	24.45	23.40	14.54	14.56	14.90

File S5. Values of ratio to no-treatment, Ct, delta Ct, and *p* values in unpaired t test in Figure 4.

Model	Gene	Treatment	Ct Value					
OD-BRE-0438	*HPRT1*	No-treatment	24.275	24.019	23.960	24.181	23.995	
		Fulvestrant	24.392	23.970	24.269	23.889	24.143	
	FGF1	No-treatment	36.592	35.080	34.167	35.504	34.661	
		Fulvestrant	34.321	34.309	34.912	33.680	35.319	
	FGF7	No-treatment	34.143	33.522	32.939	33.576	33.825	
		Fulvestrant	33.300	33.910	32.303	33.657	33.208	
	FGF8	No-treatment	33.506	33.354	33.471	33.560	33.115	
		Fulvestrant	32.847	32.558	32.403	30.977	31.751	
	FGF10	No-treatment	24.823	27.104	30.926	27.673	27.884	
		Fulvestrant	26.366	24.976	26.781	28.081	25.006	
ST2056	*HPRT1*	No-treatment	23.960	23.893	23.886	23.899	23.862	23.991
		Elacestrant	24.340	24.126	24.076	24.164	24.054	24.099
	FGF10	No-treatment	31.908	31.367	31.828	32.544	32.316	30.668
		Elacestrant	29.155	28.152	28.269	28.230	29.701	29.241
	FGF17	No-treatment	35.164	35.423	34.473	34.623	34.275	34.706
		Elacestrant	34.294	34.126	34.767	35.962	33.866	34.573
ST2535	*HPRT1*	No-treatment	22.699	22.741	22.742	22.785	22.692	22.729
		Elacestrant	22.999	22.987	22.957	23.135	22.927	23.042
	FGF10	No-treatment	30.457	31.511	31.835	31.021	30.648	31.198
		Elacestrant	29.000	29.060	29.396	28.836	29.900	30.045
	FGF17	No-treatment	34.195	34.343	34.015	33.837	34.659	34.352
		Elacestrant	33.273	33.282	32.680	33.812	34.064	33.604

Model	Gene	Treatment	Delta Ct Value						Unpaired t test
OD-BRE-0438	*FGF1*	No-treatment	12.317	11.061	10.206	11.323	10.666		0.1270
		Fulvestrant	9.929	10.338	10.643	9.791	11.176		
	FGF7	No-treatment	9.868	9.503	8.979	9.395	9.830		0.3524
		Fulvestrant	8.908	9.939	8.034	9.769	9.064		
	FGF8	No-treatment	9.231	9.335	9.511	9.379	9.120		0.0016
		Fulvestrant	8.455	8.588	8.134	7.088	7.608		
	FGF10	No-treatment	0.548	3.085	6.966	3.492	3.889		0.2467
		Fulvestrant	1.974	1.006	2.512	4.192	0.862		
ST2056	*FGF10*	No-treatment	7.948	7.474	7.942	8.645	8.454	6.677	<0.0001
		Elacestrant	4.815	4.026	4.193	4.066	5.647	5.142	
	FGF17	No-treatment	11.204	11.530	10.587	10.724	10.413	10.715	0.2676
		Elacestrant	9.954	10.000	10.691	11.798	9.812	10.474	
ST2535	*FGF10*	No-treatment	7.759	8.771	9.093	8.236	7.956	8.469	<0.0001
		Elacestrant	6.001	6.072	6.439	5.701	6.973	7.002	
	FGF17	No-treatment	11.497	11.602	11.273	11.052	11.967	11.623	0.0010
		Elacestrant	10.274	10.294	9.723	10.677	11.137	10.561	

Model	Gene	Treatment	Ratio to No-Treatment					
OD-BRE-0438	*FGF1*	No-treatment	0.44	1.04	1.88	0.87	1.37	
		Fulvestrant	2.28	1.71	1.39	2.50	0.96	
	FGF7	No-treatment	0.78	1.01	1.45	1.09	0.80	
		Fulvestrant	1.52	0.75	2.79	0.84	1.37	
	FGF8	No-treatment	1.06	0.99	0.87	0.96	1.15	
		Fulvestrant	1.82	1.66	2.27	4.68	3.27	
	FGF10	No-treatment	8.27	1.43	0.10	1.08	0.82	
		Fulvestrant	3.08	6.02	2.12	0.66	6.65	
ST2056	*FGF10*	No-treatment	0.94	1.30	0.94	0.58	0.66	2.27
		Elacestrant	8.24	14.22	12.67	13.84	4.63	6.56
	FGF17	No-treatment	0.79	0.63	1.21	1.10	1.37	1.11
		Elacestrant	1.88	1.82	1.13	0.52	2.07	1.31
ST2535	*FGF10*	No-treatment	1.54	0.76	0.61	1.11	1.34	0.94
		Elacestrant	5.20	4.95	3.84	6.41	2.65	2.60
	FGF17	No-treatment	1.00	0.93	1.17	1.37	0.72	0.92
		Elacestrant	2.34	2.31	3.43	1.77	1.29	1.92

File S6a. The original image of uncropped Western blot of phosphorylation of FGFR (Tyr653/654) in Figure S1.

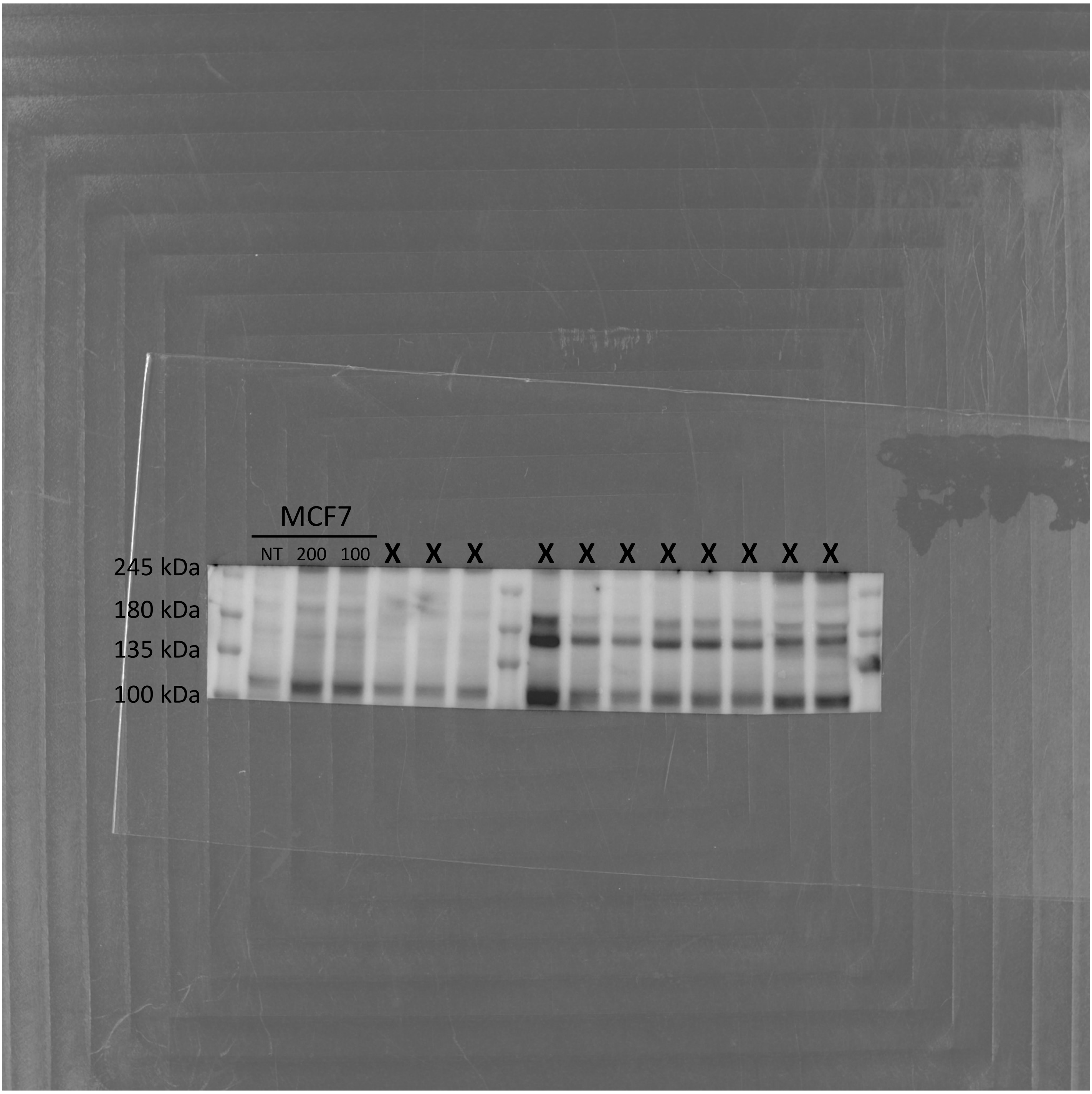

WIDE-VIEW^{TM} Prestained Protein Size Marker Ⅲ (FUJIFILM Wako, Osaka, Japan) served as molecular weight marker.
NT: no-treatment
200: treatment with 25 ng/mL bFGF + 200 ng/mL FGF10 for 30 min
100: treatment with 25 ng/mL bFGF + 100 ng/mL FGF10 for 30 min

File S6b. The original image of uncropped Western blot of phosphorylation of FGFR (Tyr653/654) in Figure S1

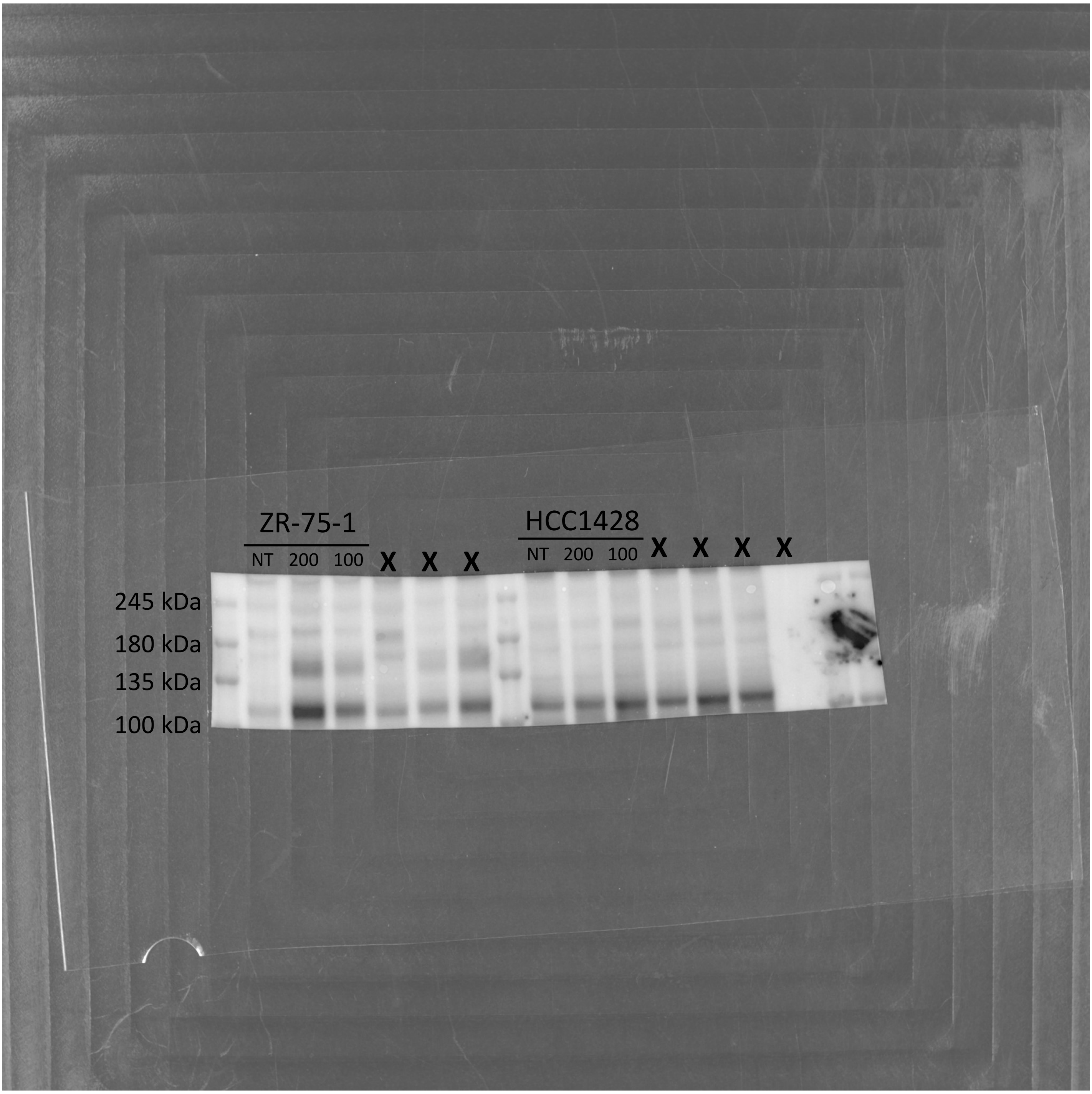

WIDE-VIEW^{TM} Prestained Protein Size Marker Ⅲ (FUJIFILM Wako, Osaka, Japan) served as molecular weight marker.

NT: no-treatment

200: treatment with 25 ng/mL bFGF + 200 ng/mL FGF10 for 30 min

100: treatment with 25 ng/mL bFGF + 100 ng/mL FGF10 for 30 min

File S6c. The original image of uncropped Western blot of phosphorylation of FGFR substrate 2 (FRS2)-α (Tyr436) in Figure S1

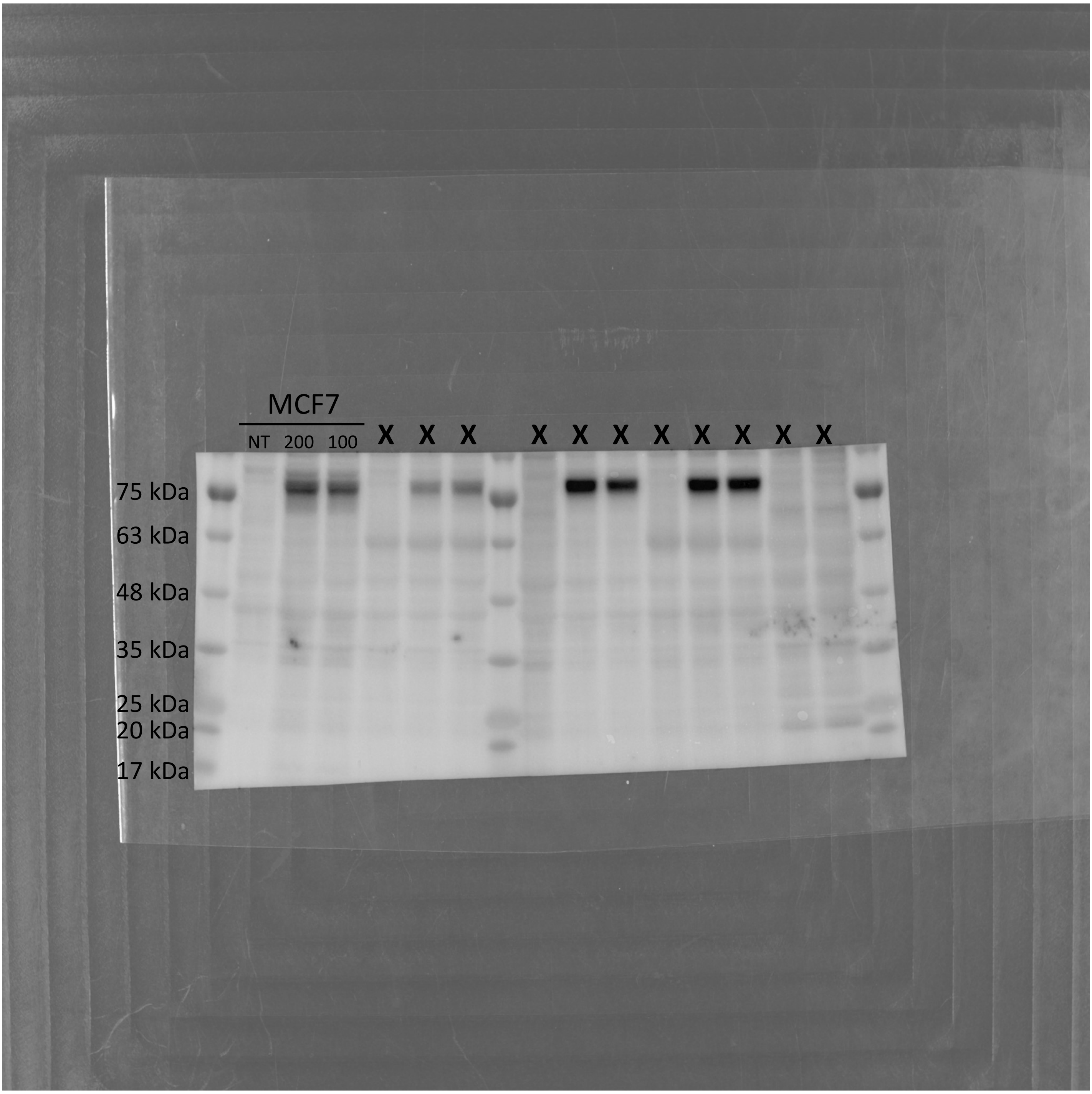

WIDE-VIEW™ Prestained Protein Size Marker Ⅲ (FUJIFILM Wako, Osaka, Japan) served as molecular weight marker.

NT: no-treatment

200: treatment with 25 ng/mL bFGF + 200 ng/mL FGF10 for 30 min

100: treatment with 25 ng/mL bFGF + 100 ng/mL FGF10 for 30 min

File S6d. The original image of uncropped Western blot of phosphorylation of FGFR substrate 2 (FRS2)-α (Tyr436) in Figure S1

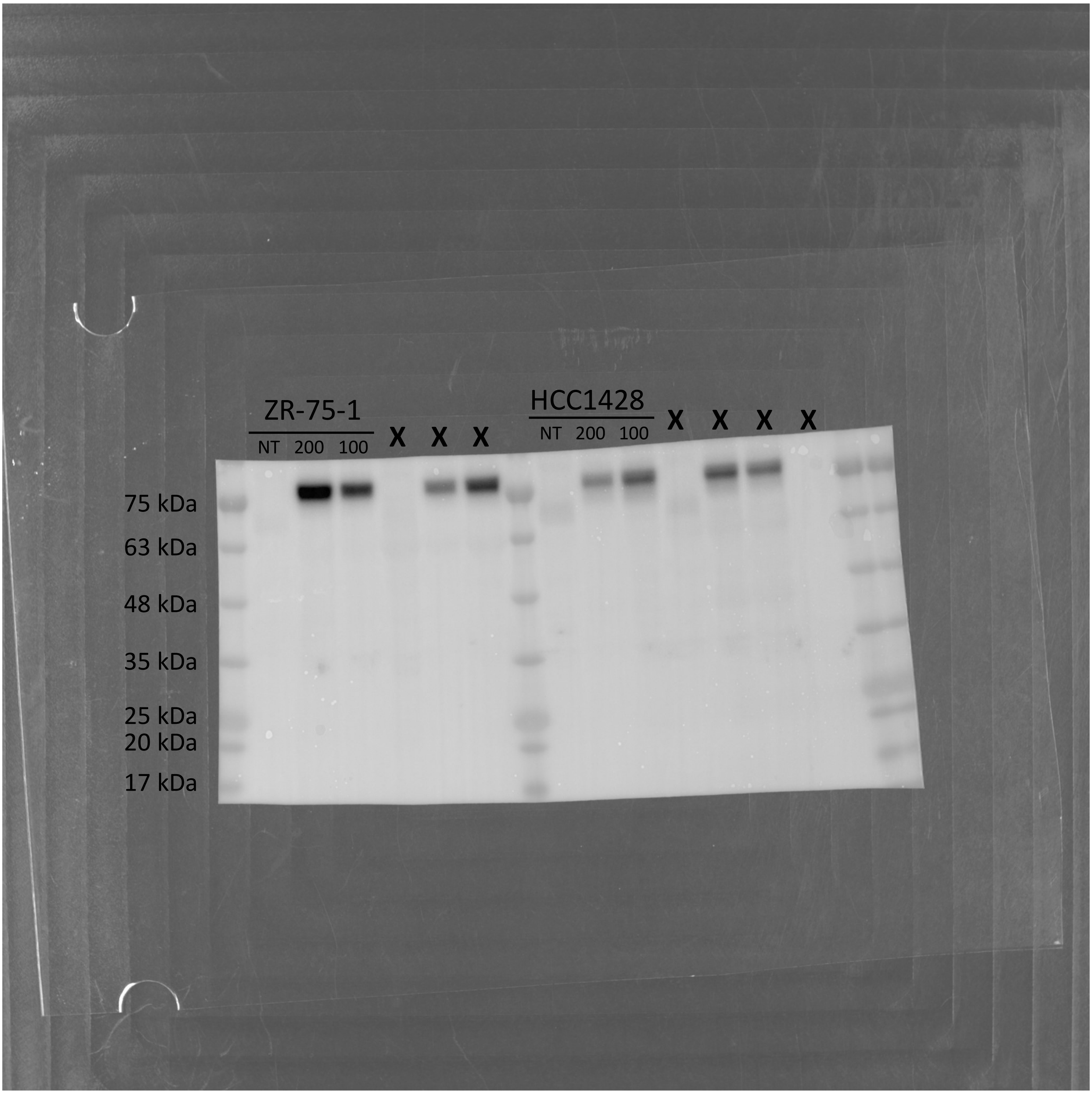

WIDE-VIEW^{TM} Prestained Protein Size Marker Ⅲ (FUJIFILM Wako, Osaka, Japan) served as molecular weight marker.

NT: no-treatment

200: treatment with 25 ng/mL bFGF + 200 ng/mL FGF10 for 30 min

100: treatment with 25 ng/mL bFGF + 100 ng/mL FGF10 for 30 min

File S6e. The original image of uncropped Western blot of phosphorylation of ERK1/2 (Thr202/Tyr204) in Figure S1

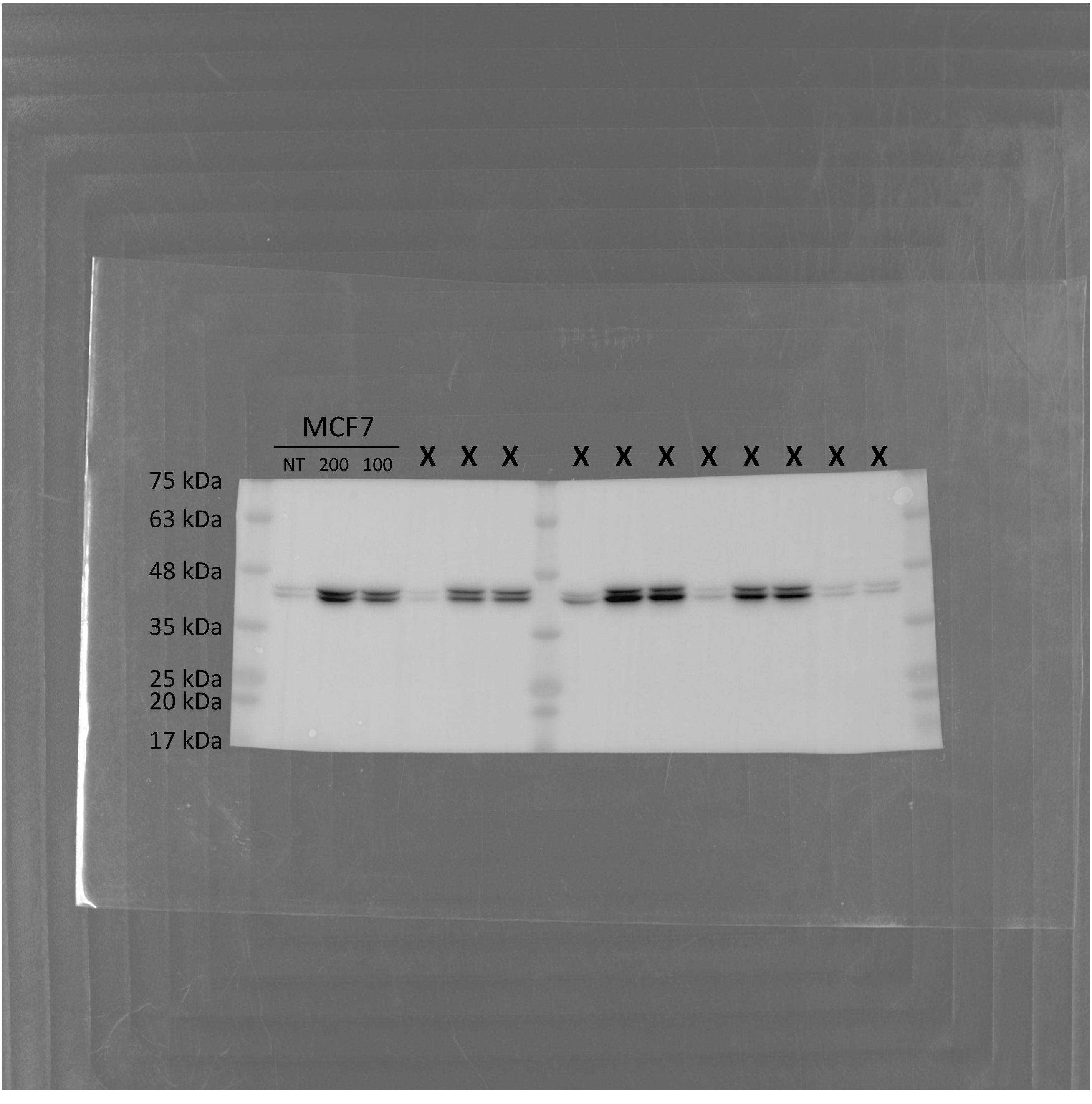

WIDE-VIEW™ Prestained Protein Size Marker Ⅲ (FUJIFILM Wako, Osaka, Japan) served as molecular weight marker.

NT: no-treatment

200: treatment with 25 ng/mL bFGF + 200 ng/mL FGF10 for 30 min

100: treatment with 25 ng/mL bFGF + 100 ng/mL FGF10 for 30 min

File S6f. The original image of uncropped Western blot of phosphorylation of ERK1/2 (Thr202/Tyr204) in Figure S1

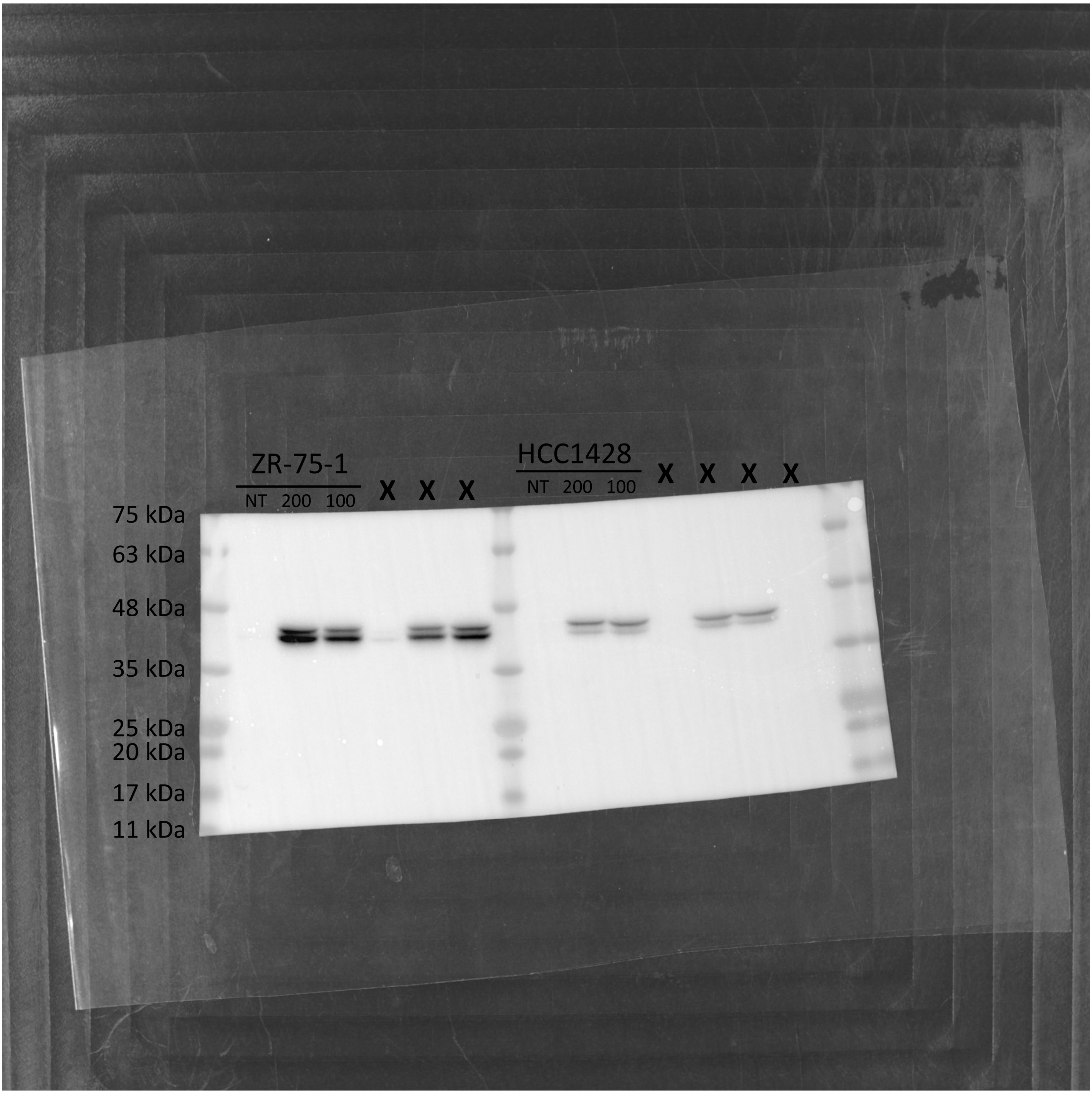

WIDE-VIEW™ Prestained Protein Size Marker Ⅲ (FUJIFILM Wako, Osaka, Japan) served as molecular weight marker.

NT: no-treatment

200: treatment with 25 ng/mL bFGF + 200 ng/mL FGF10 for 30 min

100: treatment with 25 ng/mL bFGF + 100 ng/mL FGF10 for 30 min

File S6g. The original image of uncropped Western blot of β-actin as loading control in Figure S1

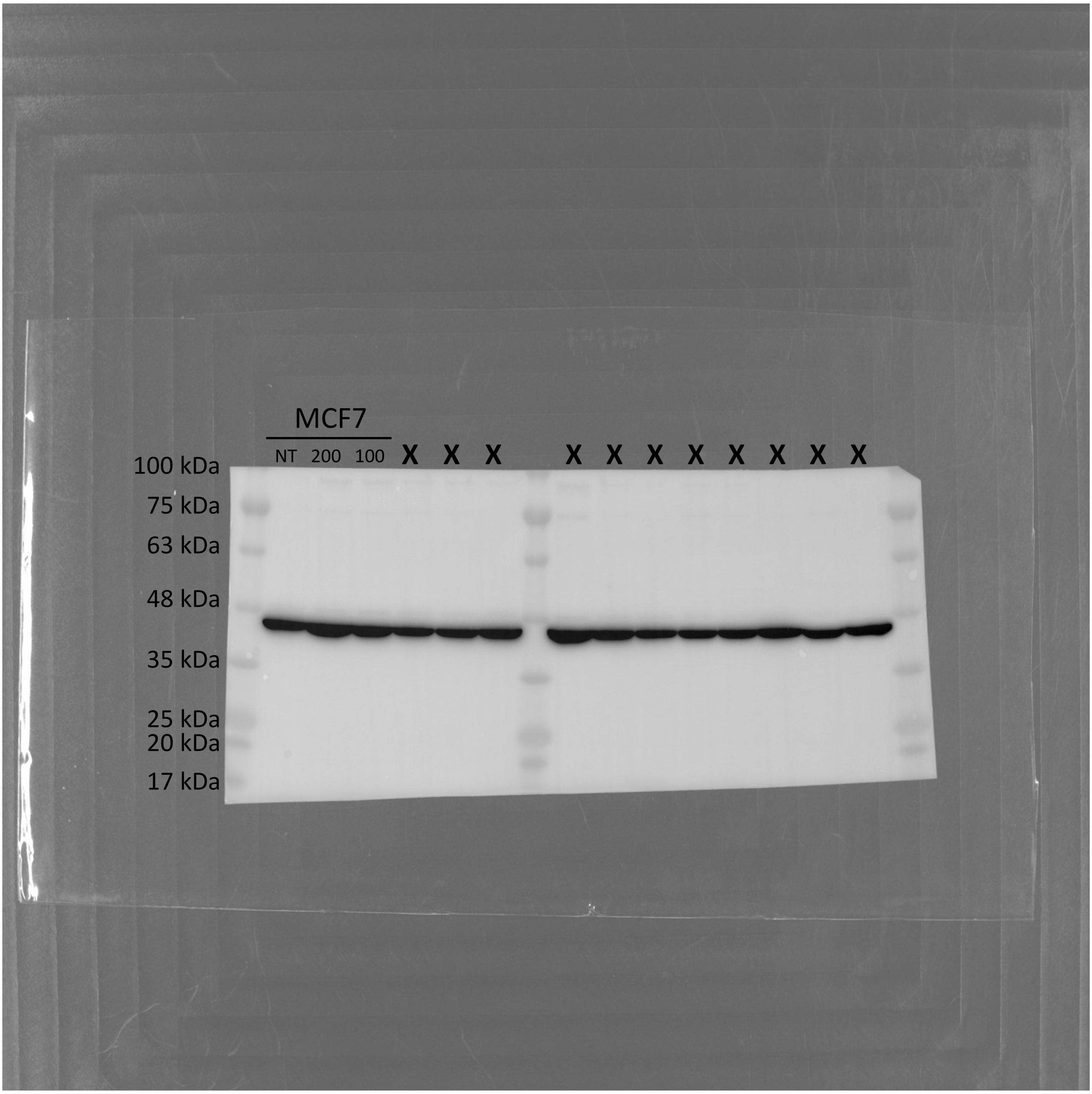

WIDE-VIEW^{TM} Prestained Protein Size Marker Ⅲ (FUJIFILM Wako, Osaka, Japan) served as molecular weight marker.

NT: no-treatment

200: treatment with 25 ng/mL bFGF + 200 ng/mL FGF10 for 30 min

100: treatment with 25 ng/mL bFGF + 100 ng/mL FGF10 for 30 min

File S6h. The original image of uncropped Western blot of β-actin as loading control in Figure S1

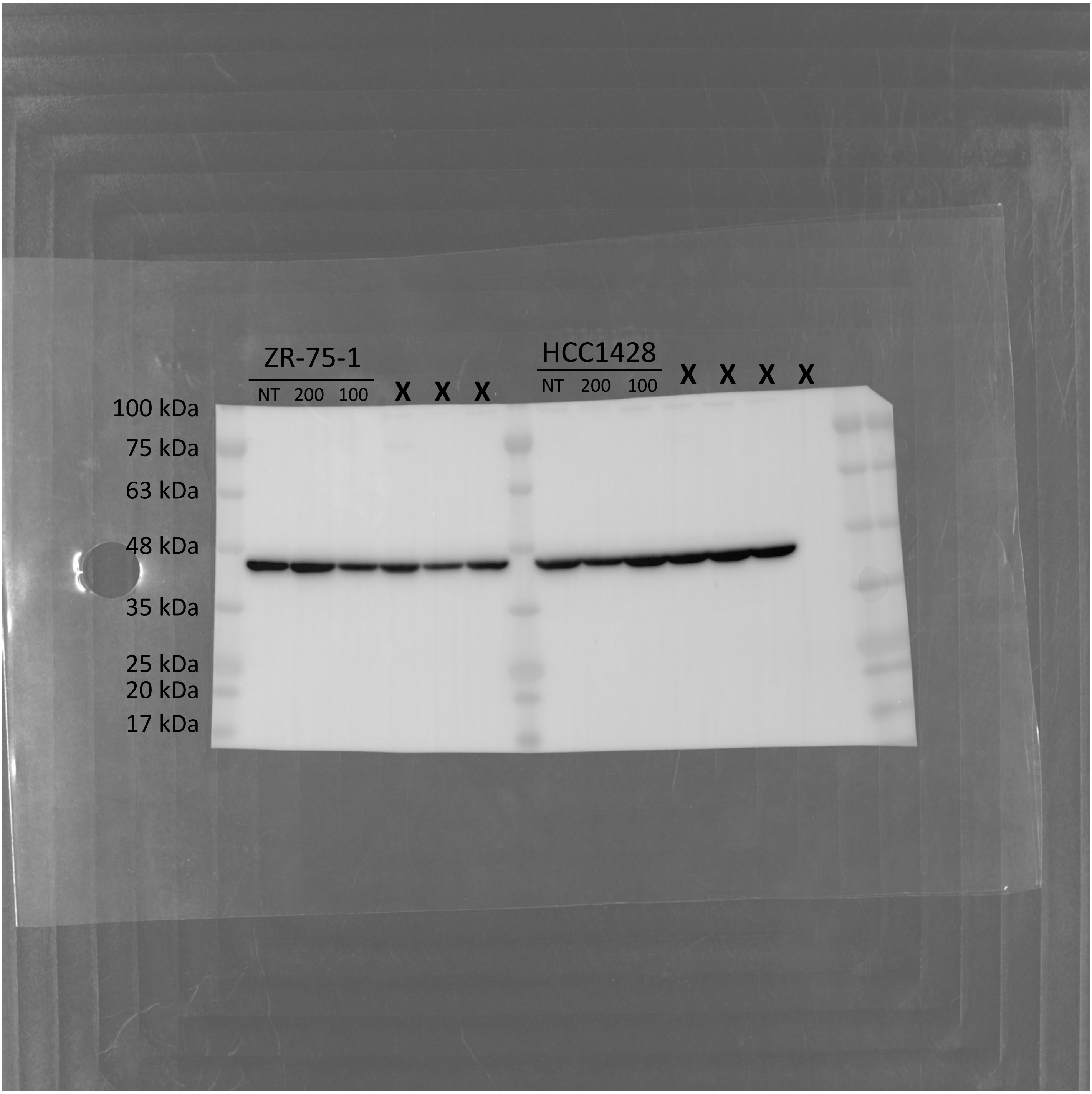

WIDE-VIEW^{TM} Prestained Protein Size Marker Ⅲ (FUJIFILM Wako, Osaka, Japan) served as molecular weight marker.

NT: no-treatment

200: treatment with 25 ng/mL bFGF + 200 ng/mL FGF10 for 30 min

100: treatment with 25 ng/mL bFGF + 100 ng/mL FGF10 for 30 min

File S7. Values of band intensities in Figure S1b.

Band	Cell Line	Treatment	Band Intensity	Intensity Ratio to β-actin	Ratio to No-Treatment
P-FGFR2	MCF7	NT	89180601	0.47	1.00
		200	124885326	0.60	1.28
		100	111602555	0.64	1.36
	ZR-75-1	NT	54887316	0.33	1.00
		200	139974591	0.77	2.33
		100	100909209	0.73	2.21
	HCC1428	NT	102451673	0.59	1.00
		200	101252443	0.73	1.24
		100	133691674	0.81	1.37
P-FRS2	MCF7	NT	87245968	0.46	1.00
		200	168250253	0.80	1.74
		100	153369156	0.88	1.91
	ZR-75-1	NT	54625105	0.33	1.00
		200	185022491	1.02	3.09
		100	137732123	1.00	3.03
	HCC1428	NT	61325684	0.36	1.00
		200	100979764	0.73	2.03
		100	139899908	0.85	2.36
P-ERK1/2	MCF7	NT	78419109	0.41	1.00
		200	154631548	0.74	1.80
		100	127308146	0.73	1.78
	ZR-75-1	NT	21145131	0.13	1.00
		200	153034178	0.84	6.46
		100	130908214	0.95	7.31
	HCC1428	NT	24725313	0.14	1.00
		200	77203216	0.56	4.00
		100	78568519	0.48	3.43
β-actin	MCF7	NT	189332656		
		200	209466191		
		100	173478245		
	ZR-75-1	NT	166543313		
		200	181436773		
		100	138073639		
	HCC1428	NT	172327041		
		200	138855971		
		100	164239866		

NT: no-treatment
200: treatment with 25 ng/mL bFGF + 200 ng/mL FGF10 for 30 min
100: treatment with 25 ng/mL bFGF + 100 ng/mL FGF10 for 30 min

File S8. Values of ratio to no-treatment, Ct, delta Ct, and *p* values in unpaired t test in Figure S2.

Model	Gene	Treatment	Ct Value					
OD-BRE-0438	*HPRT1*	No-treatment	24.275	24.019	23.960	24.181	23.995	
		Fulvestrant	24.392	23.970	24.269	23.889	24.143	
	FGFR1	No-treatment	25.783	25.587	25.461	26.092	25.382	
		Fulvestrant	26.060	25.793	26.051	25.490	25.708	
	FGFR2	No-treatment	25.367	24.361	25.724	24.713	24.884	
		Fulvestrant	25.271	25.003	24.916	25.363	24.541	
	FGFR3	No-treatment	28.327	27.878	29.185	28.017	28.104	
		Fulvestrant	27.901	28.247	27.548	27.778	27.452	
ST2056	*HPRT1*	No-treatment	23.960	23.893	23.886	23.899	23.862	23.991
		Elacestrant	24.340	24.126	24.076	24.164	24.054	24.099
	FGFR1	No-treatment	25.354	25.419	25.497	25.483	25.570	25.715
		Elacestrant	25.813	25.142	25.689	25.800	25.744	25.543
	FGFR2	No-treatment	25.643	26.027	26.029	26.145	26.351	26.279
		Elacestrant	26.782	26.557	26.350	26.690	26.717	27.016
	FGFR3	No-treatment	26.630	27.289	27.317	27.155	27.255	27.782
		Elacestrant	26.999	26.396	27.006	26.755	26.629	27.314
ST2535	*HPRT1*	No-treatment	22.699	22.741	22.742	22.785	22.692	22.729
		Elacestrant	22.999	22.987	22.957	23.135	22.927	23.042
	FGFR1	No-treatment	23.452	23.627	23.752	23.919	23.584	23.670
		Elacestrant	23.426	23.317	23.590	23.113	23.269	23.222
	FGFR2	No-treatment	24.894	24.965	25.359	25.253	26.162	25.440
		Elacestrant	25.020	25.219	24.638	26.122	24.711	24.829
	FGFR3	No-treatment	24.705	24.944	25.096	25.527	25.029	25.071
		Elacestrant	26.424	26.133	26.250	25.615	25.955	26.041

Model	Gene	Treatment	Delta Ct Value						Unpaired t test
OD-BRE-0438	*FGFR1*	No-treatment	1.508	1.568	1.501	1.911	1.386		0.3009
		Fulvestrant	1.668	1.823	1.781	1.601	1.565		
	FGFR2	No-treatment	1.092	0.342	1.763	0.532	0.889		0.9055
		Fulvestrant	0.879	1.033	0.646	1.474	0.397		
	FGFR3	No-treatment	4.052	3.858	5.224	3.836	4.109		0.1165
		Fulvestrant	3.509	4.277	3.279	3.889	3.308		
ST2056	*FGFR1*	No-treatment	1.394	1.526	1.611	1.584	1.708	1.724	0.3381
		Elacestrant	1.473	1.017	1.613	1.636	1.690	1.443	
	FGFR2	No-treatment	1.683	2.134	2.143	2.246	2.489	2.288	0.0242
		Elacestrant	2.442	2.431	2.274	2.526	2.663	2.916	
	FGFR3	No-treatment	2.670	3.396	3.431	3.256	3.393	3.792	0.0117
		Elacestrant	2.659	2.271	2.930	2.592	2.575	3.215	
ST2535	*FGFR1*	No-treatment	0.754	0.886	1.010	1.135	0.892	0.941	0.0001
		Elacestrant	0.427	0.330	0.633	-0.023	0.342	0.180	
	FGFR2	No-treatment	2.196	2.224	2.617	2.468	3.469	2.711	0.0817
		Elacestrant	2.021	2.231	1.681	2.987	1.784	1.787	
	FGFR3	No-treatment	2.006	2.204	2.354	2.743	2.337	2.342	0.0013
		Elacestrant	3.425	3.145	3.293	2.480	3.028	2.999	

Model	Gene	Treatment	Ratio to No-Treatment					
OD-BRE-0438	*FGFR1*	No-treatment	1.05	1.01	1.05	0.79	1.14	
		Fulvestrant	0.94	0.84	0.87	0.98	1.01	
	FGFR2	No-treatment	0.89	1.50	0.56	1.31	1.02	
		Fulvestrant	1.03	0.93	1.21	0.68	1.44	
	FGFR3	No-treatment	1.12	1.28	0.50	1.30	1.08	
		Fulvestrant	1.63	0.96	1.91	1.26	1.88	
ST2056	*FGFR1*	No-treatment	1.15	1.05	0.99	1.00	0.92	0.91
		Elacestrant	1.09	1.49	0.99	0.97	0.93	1.11
	FGFR2	No-treatment	1.40	1.02	1.01	0.94	0.80	0.92
		Elacestrant	0.82	0.83	0.93	0.78	0.71	0.59
	FGFR3	No-treatment	1.57	0.95	0.93	1.05	0.95	0.72
		Elacestrant	1.58	2.07	1.31	1.66	1.68	1.08
ST2535	*FGFR1*	No-treatment	1.13	1.04	0.95	0.87	1.03	1.00
		Elacestrant	1.42	1.52	1.23	1.94	1.51	1.69
	FGFR2	No-treatment	1.34	1.31	1.00	1.11	0.55	0.93
		Elacestrant	1.51	1.30	1.91	0.77	1.78	1.77
	FGFR3	No-treatment	1.25	1.09	0.98	0.75	1.00	0.99
		Elacestrant	0.47	0.57	0.51	0.90	0.62	0.63

File S9. Individual Ct values in Table S2.

Cell Line	Gene	Ct Value		
MCF7	*FGFR1*	29.633	29.426	28.841
	FGFR2	32.105	32.124	31.512
	FGFR3	29.268	29.202	28.962
	HPRT1	28.923	28.579	27.965
ZR-75-1	*FGFR1*	26.191	26.092	27.002
	FGFR2	30.085	29.756	30.435
	FGFR3	30.028	29.887	30.350
	HPRT1	30.213	30.130	30.702
HCC1428	*FGFR1*	29.266	29.372	28.874
	FGFR2	28.430	28.843	28.673
	FGFR3	32.127	32.331	32.749
	HPRT1	29.356	29.568	29.907

File S10. Tumor volume in each mouse before treatment period.

Figure	Model	Treatment	N	Tumor volume (mm^3) in each mouse before treatment period						
										Average
Figure 1a	OD-BRE-0438	NT	2	78.9	136.0	-	-	-	-	107.5
		Palb+Fulv	3	72.6	82.7	130.3	-	-	-	95.2
	OD-BRE-0704	NT	2	218.0	106.1	-	-	-	-	162.1
		Palb+Fulv	3	208.0	103.7	112.4	-	-	-	141.4
	OD-BRE-0450	NT	2	236.8	88.3	-	-	-	-	162.6
		Palb+Fulv	3	222.0	114.0	67.5	-	-	-	134.5
	OD-BRE-0188	NT	2	82.2	203.0	-	-	-	-	142.6
		Palb+Fulv	3	94.1	89.7	193.1	-	-	-	125.6
	IM-BRE-556	NT	2	146.7	118.5	-	-	-	-	132.6
		Palb+Fulv	3	142.7	163.8	113.7	-	-	-	140.1
Figure 2a	OD-BRE-0438	NT	5	128.4	134.1	145.7	158.7	190.4	-	151.5
		Tasur	5	132.8	137.4	152.3	152.8	189.9	-	153.0
	OD-BRE-0704	NT	2	218.0	106.1	-	-	-	-	162.1
		Tasur	3	226.5	120.8	167.7	-	-	-	171.7
	OD-BRE-0450	NT	3	170.6	204.4	276.4	-	-	-	217.1
		Tasur	3	166.3	209.6	294.2	-	-	-	223.4
	OD-BRE-0188	NT	2	152.1	179.4	-	-	-	-	165.8
		Tasur	2	141.3	181.9	-	-	-	-	161.6
	IM-BRE-556	NT	2	146.7	118.5	-	-	-	-	132.6
		Tasur	3	141.1	94.5	199.8	-	-	-	145.1
Figure 2b	OD-BRE-0438 after Palb+Fulv	NT	6	147.8	207.5	231.4	280.3	291.0	510.5	278.1
		Tasur	5	178.8	223.0	242.3	273.4	389.6	-	261.4
	OD-BRE-0704 after Palb+Fulv	NT	6	135.1	298.5	73.2	97.6	147.1	157.0	151.4
		Tasur	6	105.4	200.6	93.2	213.9	123.1	192.9	154.8
	OD-BRE-0450 after Palb+Fulv	NT	6	104.1	116.7	107.9	55.2	151.2	148.0	113.8
		Tasur	5	195.9	192.7	87.8	125.2	71.2	-	134.6
	OD-BRE-0188 after Palb+Fulv	NT	5	90.1	52.7	101.9	53.5	129.6	-	85.6
		Tasur	6	128.1	111.4	67.1	197.4	101.0	94.2	116.5
	IM-BRE-556 after Palb+Fulv	NT	5	129.5	348.1	116.2	181.3	355.3	-	226.1
		Tasur	5	114.4	272.5	206.6	315.2	179.5	-	217.6
Figure 5a	OD-BRE-0438	NT	5	108.9	127.4	134.7	163.9	204.6	-	147.9
		Tasur	5	109.1	123.3	143.7	169.8	197.2	-	148.6
		Fulv	5	98.1	122.7	144.1	169.2	209.3	-	148.7
		Tasur+Fulv	5	102.8	120.3	133.4	175.8	215.5	-	149.6
Figure 5b	ST2056	NT	6	175.9	254.7	250.5	419.9	242.4	165.9	251.6
		Tasur	6	247.8	239.8	251.5	154.4	466.4	215.8	262.6
		Elac	6	232.4	311.0	176.8	212.6	229.5	461.9	270.7
		Tasur+Elac	6	202.5	273.5	226.2	285.8	164.7	359.2	252.0
Figure 5c	ST2535	NT	6	216.8	341.5	182.8	240.2	253.3	151.4	231.0
		Tasur	6	216.1	361.4	181.5	238.2	226.6	112.3	222.7
		Elac	5	254.9	151.2	219.5	120.4	237.0	-	196.6
		Tasur+Elac	5	225.9	314.1	163.0	225.7	336.4	-	253.0

File S11. Passage number of PDX in each experiment.

Figure	Model	Passage Number
Figure 1a	OD-BRE-0438	P8
	OD-BRE-0704	P10
	OD-BRE-0450	P13
	OD-BRE-0188	P7
	IM-BRE-556	P6
Figure 1b	OD-BRE-0438	P8
	OD-BRE-0704	P10
	OD-BRE-0450	P14
	OD-BRE-0188	P8
	IM-BRE-556	P5
Figure 2a	OD-BRE-0438	P12
	OD-BRE-0704	P10
	OD-BRE-0450	P17
	OD-BRE-0188	P9
	IM-BRE-556	P6
Figure 2b	OD-BRE-0438	P8
	OD-BRE-0704	P10
	OD-BRE-0450	P14
	OD-BRE-0188	P8
	IM-BRE-556	P5
Figure 4a	OD-BRE-0438	P9
Figure 4b	ST2056	P7
Figure 4c	ST2535	P11
Figure 5a	OD-BRE-0438	P9
Figure 5b	ST2056	P11
Figure 5c	ST2535	P7
Figure S2a	OD-BRE-0438	P9
Figure S2b	ST2056	P7
Figure S2c	ST2535	P11
Table S1	OD-BRE-0438 (No-treatment)	P9
	OD-BRE-0438 (Palb+Fulv for 2 weeks)	P8
	OD-BRE-0704	P10
	OD-BRE-0450	P13
	OD-BRE-0188	P8
	IM-BRE-556	P5
